# Back to the Roots—An Overview of the Chemical Composition and Bioactivity of Selected Root-Essential Oils

**DOI:** 10.3390/molecules26113155

**Published:** 2021-05-25

**Authors:** Karin Lunz, Iris Stappen

**Affiliations:** Department of Pharmaceutical Sciences, University of Vienna, 1090 Vienna, Austria; a00608458@unet.univie.ac.at

**Keywords:** *Angelica archangelica*, *Armoracia rusticana*, *Carlina* sp., *Chrysopogon zizanioides*, *Coleus forskohlii*, *Inula helenium*, *Sassafras albidum*, *Saussurea costus*, *Valeriana officinalis*, roots, essential oils

## Abstract

Since ancient times, plant roots have been widely used in traditional medicine for treating various ailments and diseases due to their beneficial effects. A large number of studies have demonstrated that—besides their aromatic properties—their biological activity can often be attributed to volatile constituents. This review provides a comprehensive overview of investigations into the chemical composition of essential oils and volatile components obtained from selected aromatic roots, including *Angelica archangelica*, *Armoracia rusticana*, *Carlina* sp., *Chrysopogon zizanioides*, *Coleus forskohlii*, *Inula helenium*, *Sassafras albidum*, *Saussurea costus*, and *Valeriana officinalis.* Additionally, their most important associated biological impacts are reported, such as anticarcinogenic, antimicrobial, antioxidant, pesticidal, and other miscellaneous properties. Various literature and electronic databases—including PubMed, ScienceDirect, Springer, Scopus, Google Scholar, and Wiley—were screened and data was obtained accordingly. The results indicate the promising properties of root-essential oils and their potential as a source for natural biologically active products for flavor, pharmaceutical, agricultural, and fragrance industries. However, more research is required to further establish the mechanism of action mediating these bioactivities as well as essential oil standardization because the chemical composition often strongly varies depending on external factors.

## 1. Introduction

The use of aromatic plants by humans holds strong traditional value. However, research interest seems to increase tremendously as modern medicinal systems integrate the use of herbal remedies. Mixtures of essential oils (EOs) and volatiles represent “the essence” or odoriferous components of these plants. They have been used since ancient times as flavoring agents, as well as in the manufacture of perfumes and cosmetics, aside from pharmaceutical applications [1].

Naturally produced by aromatic plants and commonly obtained by hydro- or steam distillation, EOs are usually concentrated liquids of complex mixtures of various volatile compounds [2,3]. They are mainly constituted by terpenoids and phenylpropanoids. However, some EOs are made up of constituents of different chemical structures such as polyacetylenes or even sulfur-containing compounds [4,5,6]. In many cases, they can be characterized by up to three major components, at a relatively high concentration compared with other compounds present in trace amounts [2].

Known for their various properties, EOs are used in the preservation of foods but also as analgesic, sedative, anti-inflammatory, spasmolytic, and local anesthetic remedies [2,3]. Moreover, EOs are known to possess a wide spectrum of antibacterial, antifungal, and even anti-viral activities, as well as being capable of inhibiting the growth of drug-resistant microbial strains that are difficult to treat with conventional antibiotics [7]. The development of resistances is also an increasingly relevant issue in pest management, caused by synthetic pesticide overuse. Toxicity towards the environment, fauna as well as human health has led to a growing interest in the development of alternative pesticides. Applications of EOs have shown strong potential as insecticides, herbicides, acaricides, and nematicides [8,9,10].

However, it is important to mention that the biological activity of EOs and volatiles is strictly related to their chemical composition and may either be due to a synergism of all molecules or reflect the main molecules present at the highest levels [6]. The composition in turn depends on various factors—including genetic factors, environmental conditions, physiological, and geographic variations, among others. In particular, growth periods, seasons, and years are known to affect the accumulation of active components in roots [1,11]. Considering further that different methodologies of isolation and analysis may have been used, a comparison of EO compositions described in different studies is sometimes difficult, if not impossible [1]. Nevertheless, understanding the EO’s composition is necessary to predict its specific mode of action and therefore the possible therapeutic outcome [6].

**Figure 1:**
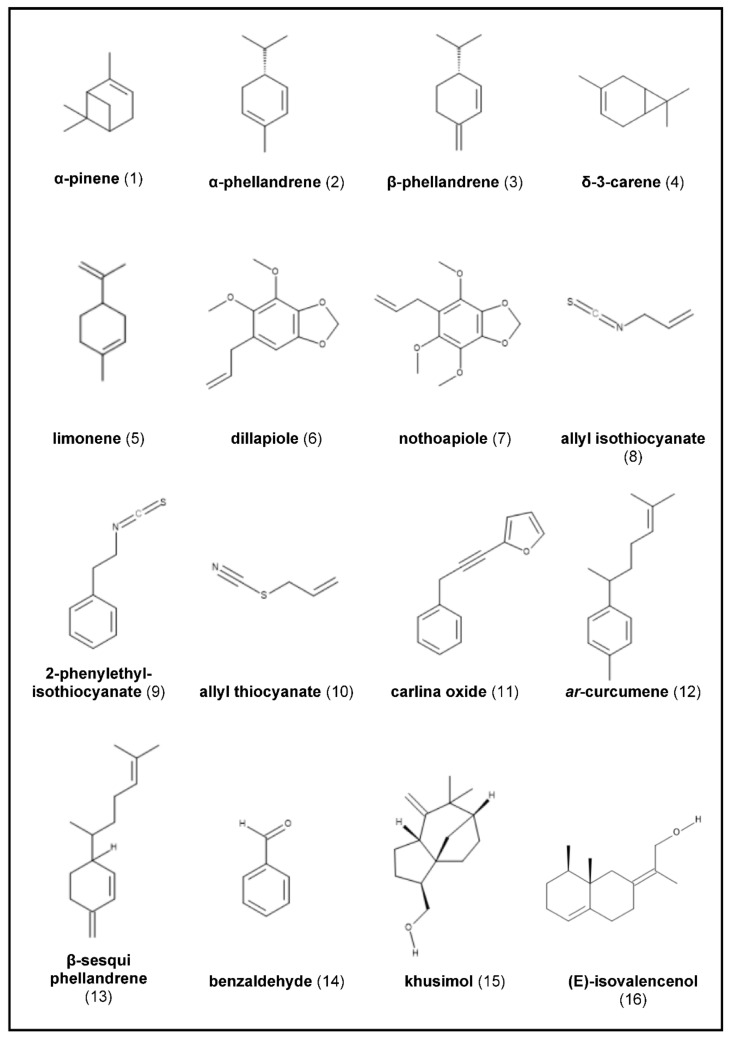

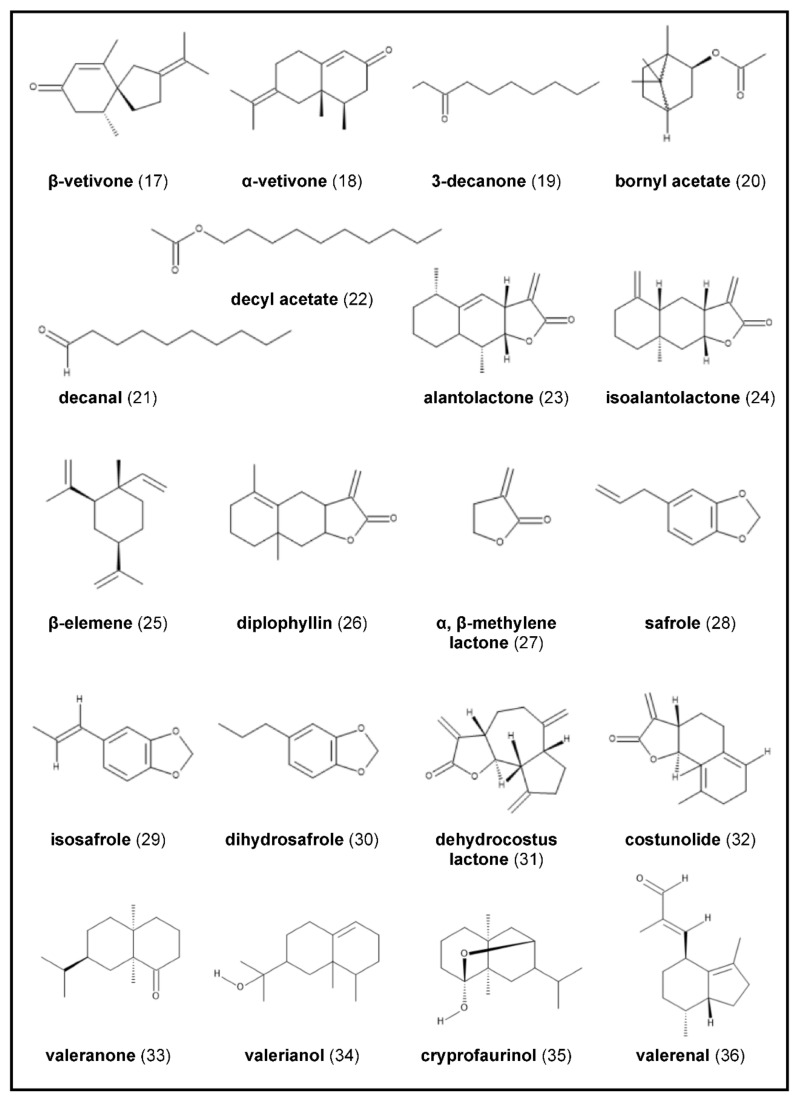
Chemical structures of principal constituents of selected aromatic roots.

In the following work, we aim to provide an overview of various biological properties associated with the EO and volatiles of nine aromatic roots, selected either due to their high impact in cosmetics and pharmacy, their interesting chemical composition, or their historical importance: *Angelica archangelica*, *Armoracia rusticana*, *Carlina* sp., *Chrysopogon zizanioides*, *Coleus forskohlii*, *Inula helenium*, *Sassafras albidum*, *Saussurea costus*, and *Valeriana officinalis*. Moreover, their main constituents—listed in Figure 1—as well as the EOs’ compositions regarding their variations are discussed.

## 2. Essential Oils from Selected Roots

### 2.1. Angelica archangelica

*Angelica archangelica* (L.) (syn. *Archangelica officinalis* HAFFM.) is an herbaceous biennial or perennial plant of the Apiaceae family, specific to the European flora, and mostly cultivated in France, Germany, Belgium, and the Netherlands [12]. The herb grows up to 1–2.5 m with a thick hallow stem, large two to three pinnate leaves, and umbels of greenish white flowers [13] (p. 77). Angelica roots are organized as a taproot system, with the main root as a storage root and numerous thin lateral roots [14].

*Angelicae radix* is approved by the German Commission E Monographs for loss of appetite and dyspeptic discomforts, such as mild spasms of the gastrointestinal tract, feeling of fullness, and flatulence [15]. Besides indigestion, *A. archangelica* roots have been used for centuries for bronchial ailments, coughs, colds, and as a urinary antiseptic in Europe [16]. Internally, the root is further applied against chronic fatigue, menstrual and obstetric complaints, as well as anorexia, and migraine [17]. As one of the most widely-used plants in traditional medicine, *A. archangelica* is well known for its aromatic properties and hence it is mainly cultivated for its roots [14]. The EO extracted from dried roots is an ingredient of various flavor formulations for alcoholic beverages, including vermouths, gins, bitters, benedictine, and chartreusetype liqueurs [18]. For therapeutical purposes, angelica root EO is used for gastrointestinal complaints such as carminative, stomachic, digestive, and antispasmodic disorders. Furthermore, it is claimed to possess depurative, diaphoretic, diuretic, expectorant, febrifuge, nervine, stimulant, and tonic properties. The EO also plays an important role in aromatherapy [19].

*Angelica archangelica* EO—produced in the roots and rhizomes—is a dark-yellow colored liquid. It is characterized by a warm, aromatic odor, with a green spicy top note and a pleasant musky dry-out [16,20]. Thus, the EO is also present in high-grade perfumes, as well as to flavor cosmetics and soaps [21]. However, since 2020, the usage of angelica root oil as a fragrance ingredient has been quantitatively restricted due to the standards of the International Fragrance Association (IFRA) [22].

The chemical composition of *A. archangelica* root oil has been extensively studied, revealing a very complex mixture [12]. Monoterpene hydrocarbons—which are responsible for the oil’s aroma—mostly account for the largest contribution to the EO content (> 60%), with α-pinene Figure 1(1), being the major or second-leading component detected in this fraction [16,23,24]. Other dominant constituents throughout are α- and β-phellandrene Figure 1(2),(3), δ-3-carene Figure 1(4), β-pinene, limonene Figure 1(5), and p-cymene [16]. Hence, monoterpene hydrocarbons are also considered as systematic markers or chemical fingerprints for *A. archangelica* root oils [23]. However, oils with a higher concentration of phellandrenes than that of pinenes are generally considered better for obtaining the angelica-like aroma [25]. *Angelica archangelica* EO further comprises decanolides (macrolide fraction), determining the musk-odor of the oil, and coumarins, i.e., osthol [16,18]. However, the relative amounts of the chemical constituents as well as the oil recoveries obtained from the rhizomes and roots have been found to vary (0.1–1%) [12]. Thus, it could be shown that the EO composition of *A. archangelica* EO roots strongly depends on the storage form prior to examination. The amount of monoterpene hydrocarbons (66.7–72.5%)—as the main constituents of uncrushed roots collected from two habitats in Lithuania—decreased 3.5–4.0 times during a storage time of about 2.5 months in pulverized or crushed-up roots. The hydrodistilled oil of roots being crushed immediately before extraction were dominated by α-pinene (15.7–19.4%), δ-3-carene (15.4–16.0%), and limonene (8.1–8.8%), or β-phellandrene (15.4%), and δ-3-carene (14.2%), depending on the harvesting localities. While parts of highly volatile monoterpene hydrocarbons evaporated from small root parts, the fraction of macrocyclic lactones and the content of coumarin osthol increased relatively two- to five-fold. Thus, the EO derived from stored pulverized or crushed roots was dominated by 15-pentadecanolide (7.2–14.9%), followed by osthol (5.3–8.8%), and 13-tridecanolide (5.4–6.1%) [16]. Due to the partial loss of the most volatile compounds, preliminary drying of *A. archangelica* rhizomes and roots prior to hydrodistillation is not recommended. Moreover, by drying some terpenes (especially α-phellandrene) become resinous. Consequently, the fresh and gently pungent notes—considered as the top fragrance notes of the oil—may be altered [12]. On the contrary, the impact of the sample preparation on the EO yield immediately prior to distillation (cutting, grinding, or powdering before or after drying) varied depending to the underground part (rhizome or root). While the sample preparation method had no influence on the EO content obtained from *A. archangelica* rhizomes by steam distillation, grinding or powdering of the roots significantly increased the EO content [20].

The extraction method is generally known to influence the composition of *A. archangelica* EO [18]. Distillation (steam distillation or hydrodistillation) has been reported to neither exhibit any noteworthy impact on the presence of the compounds nor on their concentration. However, long distillation times showed a significant influence. Thus, high molecular weight compounds (lactones, terpenes, and oxygenated sesquiterpenes) increased in their amount with distillation time at the expense of the more volatile components [26]. These results are in accordance with a study conducted on the EO of angelica roots grown in southern Brazil, isolated by hydrodistillation for 2 h, 4 h, 6 h, and 24 h. While the mono- and sesquiterpene profile did not significantly vary with distillation times ranging from 2 h to 6 h, small amounts of alcohols, lactones, and coumarins were identified in the 24 h extracted EO. According to distillation time, differences in the oil yields could also be observed, ranging from 0.2–0.8%. The main detected components were α-pinene (17.9–21.0%), δ-3-carene (12.6–13.7%), and β-phellandrene (13.2–14.5%), limonene (9.3–10.0%), and p-cymene (4.5–6.2%). Additionally, the oil was characterized by high levels of myrcene (9.4–11.8%) [18]. Acimovic and colleagues assessed the EO from roots of *A. archangelica* growing wild in Serbia. It contained α-pinene (29.7%), δ-3-carene (14.2%), and a mixture of β-phellandrene and limonene (13.2%) as the principal components. Furthermore, sabinene (6.1%), α-phellandrene (5.7%), myrcene (4.1%), p-cymene (3.8%), and trans-β-ocimene (3.6%) were detected among 59 identified constituents (99.3% of the total oil) [27]. Similarly, α-pinene (24.5%), δ-3-carene (13.8%), and β-phellandrene (10.1%), *p*-cymene (8.8%), limonene (8.4%), and sabinene (6.3%) were the most abundant components in a commercial *A. archangelica* EO from China (45 identified constituents) [23].

However, β-phellandrene—usually one of the major compounds—entirely lacked in the EO from angelica roots from central Italy, with 35 identified compounds. On the contrary, its isomer α-phellandrene (8.7%) was detected. Apart from that, the hydrodistilled EO was similar in the profile of the main components, with α-pinene (21.3%) as the dominant constituent, followed by δ-3-carene (16.5%), limonene (16.4%), and sabinene (5.1%). Other compounds yielding in amounts >5% were myrcene (5.5%), and (E)-β-ocimene (5.1%) [24,28]. Analysis of the steam distilled EO of roots from different parts of Siberia revealed a complex EO comprising 75 constituents, with β-phellandrene (30.5%) as the leading compound in a much higher concentration than previously mentioned. Apart from that, the composition of the other major constituents strongly differed except for α-pinene (23.6%), with germacrene D (9.1%), β-(*Z*)-farnesene (7.1%), and β-myrcene (4.6%) as the dominant components identified in the EO [29].

Regarding *A. archangelica subsp. archangelica var. norvegica* growing wild in France, δ-3-carene (13.0%), α-pinene (9.8%), p-mentha-2,8-dien-1-ol (8.6%), and limonene (7.3%) were found to be the principal components, whereas β-phellandrene only occurred in an amount of 2.0% of the total oil. With 15 identified constituents, this EO seemed to be less complex than oils from other studies which could be due to variations in analytical methods. According to the researchers, (*Z*)-β-ocimene, (*E*)-β-ocimene, and *cis*-piperitol could be considered as systematic markers of this species [30].

β-phellandrene and α-pinene were found to be the main constituents in the EOs of 15 populations of *A. archangelica* roots grown under identical conditions in Finland, northern Norway, and Iceland. While the chemical composition only slightly varied within the populations according to the geographical origin of the plant, it remarkably differed according to the year of harvest [21]. Furthermore, a strong variation in the EO composition—depending on different altitudes of collection—was observed by analyzing the hydrodistilled EO from angelica rhizomes grown in Western Himalaya, India. However, the chemical composition from the samples collected strongly varied from the aforementioned reports. Dillapiole (35.9–91.6%) Figure 1(6), and nothoapiole (0.1–62.8%) Figure 1(7) were reported for the first time to be the main components of *A. archangelica* rhizome oil, followed by γ-terpinene (0.3–1.1%), and myristicin (0.8–8.1%). The researchers stated that such deviation might be due to the existence of chemotypes [17]. Comparative analyses of steam distilled EOs from angelica rootles, roots, and tubers collected in France revealed the presence of 65 identified compounds, with no mentionable differences in their chemical composition. α-Pinene (22.2–28.1%) was the principal component in all underground parts (rootles, roots, tubers), followed by δ-3-carene (15.8–17.2%), while β-phellandrene (6.7–8.4%) only occurred in small amounts. Nevertheless, some specific patterns could be observed. Thus, roots contained more pinenes and yielded a higher content (0.45%) of EO than tubers (0.17%) or rootlets (0.33%)—rootlets revealing more monoterpene alcohols and tubers slightly more acetates [26]. These findings correlate with those from Letchamo et al., reporting a higher (200%) EO content in thin and thick *A. archangelica* roots compared to rhizomes. On the contrary, the largest amount of EO with 1.28% was obtained from thinner roots (1–1.5 mm diameter) followed by larger roots (1.03%) [20]. The authors concluded that regarding the higher yields of *A. archangelica* root EO, the plant should preferably be grown under conditions that favor root development over that of tuber development [26]. EO synthesis and accumulation in *A. archangelica* roots seem to correlate with the differentiation of secondary secretory ducts and therefore increase with root maturity. Only taproots exceeding 5 mm in diameter reached their full capacity of oil biosynthesis in terms of the pattern and concentration of metabolites. Thus, different developmental stages seem to cause compositional variations. Nevertheless, α-pinene (23.9–35.7%) dominated throughout, reaching the highest amount in mature roots. At this stage, α- and β-phellandrene (0.4–3.7%) also occurred in the highest amounts, which—together with pinenes—strongly influence the oil’s aroma [14].

Apart from different plant developmental stages, it is known that the content and composition of *A. archangelica* root oil significantly vary according to different greenhouse growing conditions and light intensities. Hence, the differences in the EO compositions of greenhouse- and field-produced angelica should also be considered [20]. After all, the chemical composition of *A. archangelica* EOs affects their medicinal and flavoring qualities [16]. The disadvantage of thermal degradation of most of the labile flavor compounds due to steam distillation or liquid solvent extraction [31] was avoided by supercritical fluid extraction (SFE). Thus, by optimizing SFE parameters, extracts very similar to the native aroma of angelica roots could be obtained [12]. Nykänen et al. reported that the total amount of angelica volatiles by SFE strongly depended on extraction pressure, with total amounts of volatiles ranging from 0.13% up to 0.55%. Thus, some compounds yielded up to 50 times higher than those obtained by steam distillation. The highest monoterpenoid content was acquired at 100 bar. However, reactions taking place in the presence of water at elevated temperature partly explain why some compounds are only found in the steam distilled EO [32].

Considering the traditional claim of *A. archangelica* in the treatment of various digestive problems including intestinal gas, investigations were carried out to evaluate its effects on some microorganism responsible for intestinal dysbiosis. The results indicated moderate inhibitory effects on the pathogens *Clostridium difficile, C. perfringens, Enterococcus faecalis, Eubacterium limosum, Peptostreptococcus anaerobius,* and *Candida albicans* with minimum inhibitory concentrations (MICs) of 0.25%, 0.25%, 0.13%, 0.25%, 2.25%, and 0.50% *v/v*. As expected, Gram-negative bacteria *Bacteroides fragilis* and *Escherichia coli* exerted MIC values > 4% *v/v* [24]. Due to the lack of the outer membrane, Gram-positive bacteria are expected to be more susceptible to EOs [33]. Interestingly, *A. archangelica* root oil was found to show a selectivity in the activity against the tested Gram-positive Bifidobacteria and Lactobacilli, which is particularly useful to intestinal microflora, showing MIC values > 4.0% *v*/*v*. The carminative activity of *A. archangelica* EO might be ascribed to this behavior, although further studies on the mechanism of this selectivity would be required [24]. However, results from another study were congruent regarding higher toxicity against Gram-positive strains, with *Staphylococcus aureus* (14.2 μL/mL) being more sensitive against *A. archangelica* EO than *E. coli* (28.4 μL/mL) [27]. Further, angelica root oil was found to exert moderate antiphytopathogenic fungal activity against *Fusarium* sp., *Botrytis cinerea*, and *Alternaria solani* (MICs = 300–750 (mg/mL) in vitro. However, an inhibitory effect could neither be observed against the fungus *Rhizoctonia solani* [28] nor against *Colletotrichum acutatum*, *C. fragariae*, and *C. gloesporides* [23].

Although fungitoxic investigations on isolated food-borne mold species from walnut reported *A. archangelica* EO to be ineffective against the tested mold species up to a concentration of 5.0 μL/mL, strong free radical scavenging activity was determined (IC_50_ = 1.04 μL/mL). However, an EO-based combination with α-terpineol and phenylethyl alcohol (1:1:1) was found to be more effective than the compounds tested individually, and a dose-dependent inhibition of *Penicillium citrinum*, > *Rhizopus stolonifer*, *Mucor* sp. > *Fusarium graminearum*, > *F. oxysporum* > *Aspergillus flavus* > *A. niger* and *A. alternata* in decreasing order was found. Furthermore, the mixture showed strong free radical scavenging activity. Therefore, angelica root oil is overall recommended as a safe preservative against storage molds, including aflatoxigenic *A. flavus* contamination and oxidative deterioration of walnut samples [34]. In comparative studies on the antioxidant capabilities by DPPH free radical scavenging activity and total phenolic content from 42 commonly-used EOs, angelica root oil showed an anti-oxidative activity of 17.33 ± 0.004 µmol trolox/g and a total phenolic content of 11.75 ± 0.419 (µg GAE/5 mg EO/mL EtOH) [35].

Apart from its antimicrobial and anti-oxidative properties, *A. archangelica* root oil has also been reported to exhibit significant anti-seizure activity against chemically- and electrically-induced seizures in mice. According to the researchers, the anti-seizure effect may be attributed to monoterpenes present in the oil, such as α-pinene, 3-carene, and limonene. However, monoterpenes are known to possess anticonvulsant activity due to the modulation of glutamatergic and GABAergic transmission [36,37]. Moreover, at anti-seizure doses, *A. archangelica* EO was found to produce motor impairment, probably subsequent to CNS depression caused by terpenes [36].

Fraternale and colleagues demonstrated anti-inflammatory activity of angelica root EO at low doses in vitro. By contrast, high doses (from 219.9 µg/mL) were found to be significantly cytotoxic by inducing apoptosis and necrosis in human histiocytic lymphoma cells. Thus, angelica EO may have potential as an inflammatory tool, considering the dose dependence [38].

### 2.2. Armoracia rusticana

*Armoracia rusticana* (L.) (syn. *Cochlearia armoracia*, *Armoracia lapathifolia*)—also known as horseradish—is a perennial crop belonging to the Brassicaceae family [39]. The plant is indigenous to Eastern and Northern Europe and the Mediterranean. It is also cultivated in Central Europe [40]. Horseradish has a glabrous stem up to 120 cm height and a wavy margin leaf rosette with white tetramerous flowers arranged in racemes. The long, cylindrical or tapering main root with several lateral roots reaches a length of 60 cm in loose soil. It is characterized by a brown outer skin and a fleshy white interior. If undisturbed, the root system can reach a depth of 3–4 m with a lateral spread of about 1 m [41]. The root by itself is inodorous, but it releases a short-lasting pungent, burning, intensive, and lachrymatory odor upon grating, cutting, or shredding [40,42].

*Armoracia rusticana* has been extensively used since ancient times for its nutritional value and as a culinary spice [43]. Traditionally, fresh and dried roots have been consumed as a condiment as a paste or sauce, containing grated root, acetic acid from vinegar, and salt. However, processed products including dehydrated and freeze-dried horseradish are gaining in importance [44,45]. It is further used as a preserving agent, antiseptic, and fermenter in many regions worldwide [44]. However, due to its strong organoleptic properties—which can lead to serious side effects—the use of high concentration of horseradish root is limited [46]. Moreover, *A. rusticana* is well known as a folk medicinal herb [43]. In Europe, it was traditionally used to treat gout, kidney stones, asthma, and bladder infections. Additionally, horseradish is reported as a remedy against pain associated with rheumatism, headache, and for lowering blood pressure. Due to its blood circulation-promoting and skin-warming properties, grated horseradish is also known as a home remedy for chest congestion and stiff muscles. Furthermore, it was used to prevent scurvy before vitamin C was discovered [42,44].

Belonging to the Brassicaceae family, uncrushed horseradish is rich in sulfur-containing glycosides called glucosinolates (GLS), which play an important role in the plant defensive system. Among them, sinigrin (2-propenyl glucosinolate) is the major glucosinolate in the intact roots, accounting for up to 78% of the total GLS, followed by gluconasturtiin. When the cells become disrupted by grating or chewing, GLS are hydrolyzed by the plant-based enzyme myrosinase to volatile sulfur-containing compounds. Among them, isothiocyanates (ITCs) are the dominant components, followed by nitriles and/or thiocyanates. Thus, the typical sharp taste and smell of horseradish roots is mainly attributed to sinigrin-derived allyl isothiocyanate (AITC) Figure 1(8) and gluconasturtiin-derived 2-phenylethyl isothiocyanate (PEITC) Figure 1(9) [39,43,47].

Investigations on fresh commercial *A. rusticana* roots from Serbia afforded 0.14% of hydrodistilled volatiles. GC-FID and GC-MS analysis identified 99.8% of the total oil, revealing the presence of ten different constituents. AITC (56.3%) and PEITC (37.3%) were the principal compounds, followed by allyl thiocyanate (5.0%) (ATC) (Figure 1/10). Other constituents (e.g., 3-butenyl and benzyl ITC) occurred in trace amounts under 1.0% [47]. The three main compounds were congruent with those of the EO of dried horseradish roots from China, although their amounts markedly varied. Thus, the Chinese EO was characterized by a significantly higher amount of PEITC (77.8%). On the other hand, AITC (15.9%) and ATC (2.2%) yielded comparatively lower amounts than previously reported. Additionally, n-butyl ITC, 3-butenyl ITC, n-amyl ITC, 5-hexenyl ITC, benzyl cyanide, and benzyl ITC were identified in minor concentrations [48].

Analytic results demonstrated a strong influence of the genotype on the qualitative and quantitative content of volatiles in horseradish roots, varying from 0% to 81.82%. Solid phase microextraction (SPME) revealed the presence of 15 volatile components, with predominance of isothiocyanates. Among them, AITC (64–82%) was the main aroma constituent of all samples, aside 2-phenylethylisothiocyanate (4–26%) that was formed from glucosinolate—gluconasturtin [40].

Moreover, differences between volatile compounds found in the fresh horseradish root immediately after harvesting and in the cut of roots kept at 5 °C for 12 h using SPME-GC-MS have been shown. While AITC (81.0%), 4-isothiocyanato-1-butene, and PEITC were the main volatile constituents registered immediately after harvesting, ethyl isothiocyanate dominated in cut horseradish. Most of the volatile compounds rapidly declined after cutting [43,49]. Consequently, it is stated that horseradish roots should be consumed quickly or refrigerated to minimize loss of volatile flavor compounds and retain their pungency and quality [43].

Mazza and colleagues assessed the impact of post-harvest processes such as freeze drying and dehydration on the quality and quantity of flavor components of Canadian horseradish compared with fresh sliced (1 mm thick) roots. The hydrodistilled oil comprised 10 constituents, with AITC, PEITC, vinyl acetonitrile, ATC, and 3-phenyl propionitrile as the main compounds. By comparison, the EO from processed roots lead to higher amounts and contained more AITC and less PEITC than the oil from fresh roots, with almost the same number of other volatiles. Thus, it seemed that the synthesis of volatiles during processing (dehydration and freeze drying) of horseradish might be higher than the loss, yielding volatiles that were superior in quality. Content and oil composition further significantly varied according to distillation time, decreasing in quality. However, no relevant difference between thick and thinner roots was observed [45]. While there was no significant difference between hydrodistillation (5.83%) and SFE (6.10%) in AITC output from *A. rusticana*, both methods resulted in higher extraction efficacy than water extraction (3.39%) [50].

The antimicrobial activity of horseradish oil has been evaluated and demonstrated for a broad spectrum of microorganism, in both the solid and vapor phase. In particular, AITC has demonstrated strong potential as a food-borne and natural preservative to prolong the shelf life of various foods [51]. Thus, the suppression of spoilage lactic acid bacteria by AITC treatment at 2000 mg/L was reported only recently [52]. Nedorostova et al. evaluated the antimicrobial properties of 27 EOs as a vapor phase against food-borne bacteria *E. coli*, *Listeria monocytogenes*, *S. aureus, Pseudomonas aeruginosa*, and *Salmonella enteritidis.* The vapor of horseradish EO was found to possess the strongest activity against all of the tested strains (MIC = 0.0083 µl/cm^3^) and thus showed potential in the control of food-borne bacteria pathogens [53]. Bacterial pathogens in ready-to-go vegetables could be controlled by treatment with purified AITC vapor, exerting bactericidal activity against food-borne pathogens (*S. montvideo*, streptomycin-resistant strains of *L. monocytogenes* and *E. coli*) in iceberg lettuce, apples, and tomatoes [54]. Only recently, fumigation treatment with horseradish oil as well as eight isolated ITCs showed strong potential as preservatives and antifungal agents for post-harvest tomato disease control (*B. cinerea*, *A. alternata, R. stolonifer*, and *Geotrichum candidum*) [48].

Although AITC is well known as a safe and non-toxic food-borne antimicrobial agent, its widespread application is hampered by its instability, mustard-like irritating odor, and spicy taste [55]. This characteristic odor is reported to be reducible by citrus and vanilla flavor, without reducing its bactericidal activity [56]. In this context, the in vitro stability of AITC after microencapsulation and its effect on the preservation of chilled fresh pork for 24 days has been evaluated. Compared to free AITC treatment, significantly higher stability, and consequently a more lasting antibacterial effect could be shown. Without direct food contact, the shelf life of pork tenderloin (*Musculus psoas major*) could be duplicated, and the release of irritant odor reduced. Gram-negative bacteria such as *E. coli* and *Salmonella* were more susceptible to AITC treatment than Gram-positive bacteria *L. monocytogenes* and *S. aureus*. Conclusively, AITC microencapsulation treatment demonstrated a higher level of security guarantee and convenience to use, although further studies are needed [51]. In addition to antimicrobial-associated preservation, purified AITC could also reduce decay in fruit tissue exerting pro-oxidant effects by paradoxically generating additional amounts of reactive oxygen species (ROS) to inhibit the growth and proliferation of microbial cells [55]. Thus, among others, the reduction of decay of various berry fruits (strawberries, blackberries, raspberries, blueberries) during storage has been reported [55,57]. Recent studies on the effects of encapsulated AITC (4%) in zein ultrafine fibers on postharvest quality of strawberry elucidated a successful reduction of weight loss and prolongation of shelf life of strawberry fruit for additional five days [58].

Apart from its potential as a preservative agent, horseradish EO further showed the most potent antifungal activity among 70 tested EOs in the vapor phase to control Chalkbrood disease in honey bee larvae (*Apis mellifera* L.), caused by the fungus *Ascosphaera apis* (MIC = 16 µL/L) [59].

The antimicrobial and fumigant activity of horseradish EO and extracted ITCs against food-borne and plant pathogens has been well documented, but only little is known about their antimicrobial properties against human pathogens [60]. *Armoracia rusticana* volatiles were reported to be effective against Gram-positive bacteria *S. aureus*, *S. epidermidis*, *Bacillus subtilis*, and *E. faecalis*, as well as Gram-negative bacteria *E. coli*, *Klebsiella pneumoniae*, *P. aeruginosa*, and *S. abony*, (MIC = 265–795 μg/mL) [47]. Among seven different evaluated EOs in the vapor phase, horseradish was found to exert the lowest and most consistent MICs (8.3–17 µL/L) against several strains and clinical isolates of *S. aureus*, including Methicillin-resistant strains (MRSA) [61]. Among four strains of antibiotic-resistant bacteria and three strains of normal pathogenic bacteria, multidrug-resistant *P. aeruginosa* (minimal bactericidal concentration (MBC) = 208.3 μg/mL) and *A. baumanii* (MBC = 41.7 μg/mL) were the most susceptible to isolated ITCs, with AITC and PEICT as the main active compounds. Being more effective against the tested bacteria compared with antibiotics, ITCs are suggested as antibacterial agents against antibiotic-resistant bacteria [62].

Regarding antifungal toxicity to human pathogens, *A. rusticana* volatiles exerted significant activity against the ATCC strain of *C. albicans* (MIC = 28.7μg/mL), and 20 clinical isolates of *C. albicans*, obtained from throat, tongue, skin, nasal, cervical, and vaginal swabs, and stool (MIC = 28.7 μg/mL) [47]. According to another study, horseradish EO exhibited potent fungicidal activity against *C. albicans* in the liquid and volatile phase, respectively. However, in the liquid phase the effect of the EO was more significant than its main constituents AITC and PEITC [63]. Potent concentration-dependent antifungal activity from horseradish-extracted ITCs has also been shown against pathogenic dermal fungi such as *Trichophyton rubrum*, *T. mentagrophytes*, *Microsporum canis*, and *Epidermophyton floccosum,* with MICs of 100–200 μg/mL, and minimal fungicidal concentrations (MFCs) of 200 µg/mL [64].

In terms of insecticidal properties, glucosinolate breakdown products are known for their potential as biodegradable and safe insect fumigants. It is stated that they may act on the insect respiratory system [65,66]. Thus, the high fumigant efficacy of *A. rusticana* EO and its main compound AITC are suggested to be attributed to the blockage of the spiracles of the insects by impairing breathing, which leads to death by choking [67,68]. Treatment with horseradish oil has been found to exhibit potent insecticidal activity against adult rice weevil *Sitophilus oryzae* and adult adzuki bean weevil *Callosobruchus chinensis*. Contact application and fumigation method led to 100% mortality within one day after treatment, while no mortality was observed in open containers [65]. Park et al. reported that at 1.25 µL/L air, horseradish EO caused 100% mortality to larvae of *Lycoriella ingenua* flies, decreasing dose dependently to 3.3% at 0.625 µL/L air [67]. Moreover, horseradish EO and extracted AITC have been reported as a potentially safe and environmentally benign alternative to chemical fumigants with high toxicity against four major pest species in stored products (maize weevil *S. zeamais*, lesser grain borer *Rhizopertha dominica*, *Tribolium ferrugineum*, and book louse *Liposcelis entomophila*). While the fumigation efficacy of horseradish EO was unaffected by temperature, it directly depended on the presence/absence and type of stored grain (maize > wheat > paddy). It could be shown that AITC residues in the crop were extremely low, and should unlikely be a concern for human health [50,69]. At 32 µL/L gas vapor, *A. rusticana* oil was highly effective against adults of *S. zeamais* (100%) as well as different life stages of *Plodia interpunctella* [68].

Apart from that, horseradish oil (24 h, LC_50_ = 1.54 µg/cm^2^) and AITC (2.52 µg/cm^2^) have also been found to possess high acaricidal toxicity against adults of the american house dust mite, *Dermatophagoides farina.* Being more toxic than either benzyl benzoate or dibutyl phthalate—two conventional acaricides—horseradish oil and AITC may be potential novel and effective dust mite control products. The toxicity was evaluated using contact + fumigant and vapor phase mortality bioassays [70].

Several epidemiological experiments have indicated an inverse correlation between the dietary consumption of Brassicaceae vegetables and the incidence of cancer [71]. Glucosinolates and ITCs as their degradation products are widely known for their preventive and cytotoxic properties in various cancer cell lines [71,72,73] by modulation of epigenetic targets [74]. Thus, ITCs are known for their anti-proliferative, pro-apoptotic, anti-inflammatory, anti-migratory, and anti-angiogenic effect against several cancers [74]. Among them, PEITC belongs to the key ITC compounds with multifaceted molecular mechanisms and known for anti-inflammatory effects and potential chemopreventive and anti-tumor properties [71,73,74]. As reviewed only recently, PEITC has found to exert potential anti-cancer effects, among others, against breast, colorectal, glioblastoma, lung, oral, ovarian, and prostate cancer [74]. Moreover, homogeneous PEITC-containing topical carbomer gel was able to kill squamous cell carcinoma directly, as well as to sensitize cancer cells to radiation at small concentrations (0.5% *v*/*w* PEITC). Further studies are warranted [75].

Apart from PEITC, also AITC has been studied for its anti-tumor, anti-proliferative and chemopreventive impact [76]. Thus, anti-cancer effects are reported against various cancers, such as cervical, lung, oral, ovarian, and prostate cancer [74]. Moreover, the anti-estrogenic and anti-proliferative effect of AITC-inhibited neoplastic transformation and the progression of chemically-induced mammary carcinogenesis in rats could be shown. It is stated that the putative chemopreventive mechanism is mainly due to controlling the regulation of estrogen synthesis, the expression of estrogen receptors and cell proliferative markers [77]. Only recently, a novel mechanism was found by which AITC triggered mitochondrial-dependent apoptosis via G2/M phase arrest and ROS-based ER stress in colorectal adenocarcinoma cells (HT-29 cells) [78]. Furthermore, results indicated AITC as a potential component worth further investigation in human cisplatin-resistant oral cancer. AITC significantly decreased cell viability and promoted mitochondria-dependent apoptotic pathway through AITC-enhanced activities of caspase-3 and caspase-9. Additionally, the treatment inhibited Akt/mTOR proliferation signaling [79].

In correlation with the beneficial claims of a diet containing horseradish, its anti-oxidative properties have been studied. Thereby, it could be shown that diet containing horseradish addition had no effect on the antioxidant ability of plasma and the heart in mice, suggesting that the biological attributes of this plant are not related to its antioxidant properties [72]. Assessing the antioxidant abilities of horseradish roots, a remarkable correlation between the environment conditions of plant growth and horseradish type could be shown. The results of every analytical method applied (ability to reduce Fe^3+^ ions, and inhibition of deoxiribose oxidation) indicated that horseradish volatile oil possessed stronger antioxidant properties than pure AITC [72].

### 2.3. Carlina acaulis (and Related Species)

*Carlina acaulis* L.—also known as stemless carline thistle, dwarf carline thistle or silver thistle—is a monocarpic, perennial plant from the Asteraceae family. The thistle-like shrub is distributed in Alpine regions of central and southern Europe, growing up to 2000 m in altitude [80]. The plant is characterized by a lanceolate, prickly dentate leave rosette, tubular flowers, collected in silvery-white florets with a diameter from 7 to 15 cm and an achene fruit. The taproot is thick and fleshy [13,80] (p. 149).

*Carlina acaulis* is widely recognized as an important medicinal plant in the Alpine regions of central and southern Europe [81]. The drug—referred to *Carlinae radix*—has been used since ancient times as tinctures and decoctions against various ailments [82]. It is indicated as diuretic, diaphoretic, stomachic, anthelmintic, and as a remedy against several gastrointestinal diseases (emetic, laxative). Furthermore, *C. radix* was used in the treatment of dental ailments and as a gargle against catarrh. Additionally, its veterinary use is reported. However, the main purpose of this drug until today is the external use for various skin diseases and inflammations, including wound healing. Thus, the treatment of herpetic eruptions and suppurating rashes (pyodermias) was also reported [13,82]. Nowadays, *C. acaulis* is still recognized as a remedy in the folk medicine of mountainous areas through Europe, although it lost its importance as a medicinal plant at the end of the 19th century for unclear reasons [5]. Limited effectiveness, undesirable toxicity, or its limited presence in natural habitats due to massive collection might be possible reasons [5,83]. Along with its medicinal use, *C. acaulis* is still recognized as a food plant (receptacle and roots) [84] and—together with its EO—is listed in the BELFRIT botanical list as a botanical food supplement by Italy [85], Belgium, and France [86]. However, examinations of dried commercial *C. radix* samples have convincingly demonstrated that the roots mostly belonged to *C. acanthifolia* All. instead of *C. acaulis,* as is required by the Erg. B. 6 (Appendix to German Pharmacopeia Sixth from 1941) [87,88,89]. *Carlina acanthifolia* is another widely distributed and less rare carlina species, considered as an adulterant or substitute for the root of *C. acaulis* [13,87,89]. According to organoleptical, morphological, and anatomical features, the two species are remarkably similar [87] and have traditionally been used for the same medicinal purpose [90].

Regarding the chemical composition of *C*. *radix*, reports are scarce. Literature data indicate that the roots of carlina spp. mainly comprise inulin (18–20%), flavonoids, and 1–2% of an EO [89,91,92], with a quite simple profile [88]. However, its biological activity is mostly associated with the EOs. Using Raman spectroscopy, it was possible to localize the EO reservoirs, which were mostly found in the structures of the outer layer of the root (i.e., cortex), while the inner part showed nearly no signal assigned to the oil [93].

Carlina oxide (2-(3-phenylprop-1-ynyl) furan) Figure 1(11)—a natural polyacetylene—has been found to be the outstanding principal and bioactive component of the EO through all studied samples (ranging from 91.5% to 99.0%) [5,10,81,88,91,93,94,95]. Apart from varying concentrations, the results obtained were all in accordance. EOs derived from cultivated and wild populations from either *C. acaulis* or *C. acanthifolia* were composed similarly up to a significant level [88,89].

Steam distillation of *C. acaulis* roots collected from wild in Serbia yielded 1.3% of an EO, yellow in color and characterized by a narcotic odor and pungent taste. Besides carlina oxide as the main component (97.2%), benzaldehyde (0.8%), *ar*-curcumene (0.6%) Figure 1(12), heptane (0.5%), 1-phenyl-2-propanone (0.2%), (*Z*,*E*) α-farnesene (0.2%), and β-sesquiphellandrene (0.2%) Figure 1(13) were found in amounts < 1% [91]. Similarly, carlina oxide (96.2%), benzaldehyde (0.6%) Figure 1(14), *ar*-curcumene (0.6%), and β-sesquiphellandrene (0.2%) were identified as the main components in the hydrodistilled EO from *C. acaulis* roots collected in Lublin, Poland [5].

However, the hydrodistilled EO (0.4%) of commercial *C. acaulis* root samples obtained from an Albanian accession was characterized by a comparatively higher amount of benzaldehyde (3.1%). Furthermore, carlina oxide (94.6%), and *ar*-curcumene (0.4%) were identified in the orangish colored EO, with acetophenone, benzyl methyl ketone, camphor, and carvone only occurring at trace levels [10,81]. A total of four compounds, accounting for 99.96% of the total composition, was detected in hydrodistilled *C. acaulis* root EO from another Albanian accession (0.74% yield), with carlina oxide (97.7%) as a major component. Among the minor constituents, benzaldehyde (1.5%), *ar*-curcumene (0.7%) and β-sesquiphellandrene (0.1%) were detected [95].

Analyses of the EO obtained from *C. acanthifolia* root samples growing in Eastern Serbia enabled identifying nine constituents, representing almost 100% of the total oil. The steam distilled EO was a yellow liquid when freshly isolated, with an intense narcotic odor and with carlina oxide (91.5%) detected as the predominating component. Furthermore, β-sesquiphellandrene (2.8%), α-zingiberene (2.4%), *ar*-curcumene (1.6%), and γ-curcumene (1.1%) were obtained in amounts above 1%. Other identified constituents were (*E*)-β-farnesene (0.4%), β-bisabolene (0.1%), and (*Z,E*)-farnesal (0.1%) [89]. Strong similarity was found with the EO composition of *C. acanthifolia subsp. utzka* root grown in Poland. Carlina oxide (99.0%) was the principal component, followed by α-zingiberene (0.2%), β-sesquiphellandrene (0.2%), and *ar*-curcumene (0.2%) [93].

Investigations on commercial *C. radix* drug samples from Serbia (claimed by the seller to solely comprise *C. acaulis*) revealed a hydrodistilled EO with 11 constituents, among which carlina oxide predominated outstandingly, representing 98.9% of the total oil. β-Sesquiphellandrene (0.1%), *ar*-curcumene (0.2%), and (*Z*,*E*)-α-farnesene (0.1%) were found in smaller amounts than previously reported, while α-zingiberene and other constituents were not present or occurred in trace amounts (<0.05%). However, morphological and anatomical studies undoubtedly demonstrated that the tested samples belonged to *C. acanthifolia* instead of *C. acaulis* [88].

Studies on the influence of different parameters on the phytochemical composition of carlina are scarce. Strzemski and co-workers recently reported that the composition of *C. acaulis* strongly varied according to different cultivation systems (in vitro cultures, hydroponic cultures, and field cultivation), with the amount of carlina oxide being highest in plants from the field [83]. Furthermore, it could be shown that treatment with silver ions increased the content of carlina oxide in the roots of *C. acaulis* [96].

Apart from that, using Raman spectroscopy, long-term stability of the EO of *C. acanthifolia subsp. utzka* during storage could be demonstrated. After 35 days, no change in the EO composition was observed [93]. However, since the EOs of *C. acaulis* and *C. acanthifolia* are remarkably similar in their chemical composition, it can be assumed that an adulteration or substitution of the drugs with each other would not cause significant differences in their biological activity [88].

Although *C. acaulis* is widely used in traditional medicine, its biological potential has only been little explored to date [81]. However, the possibility of reintroducing *C. acaulis*-derived extracts to phytotherapy has increased in interest and recently been extensively investigated [5]. Thus, several studies have confirmed the potential of carlina root EO and carlina oxide to inhibit and kill bacteria and fungi as compared with standard microbial drugs [97]. These findings strengthen the ethno-pharmaceutical use of carlina in the treatment of several human infections [88].

*Carlina acanthifolia* root EO was found to possess significant antibacterial activity (MIC = 2.5, 5 and 10 µL/mL), with the highest potency against Gram-positive bacteria such as *Streptococcus pyogenes, E. faecalis*, *B. subtilis*, and *S. aureus*. Further, efficacy against the Gram-negative strain *K. pneumoniae* and a potent antifungal activity against *C. albicans* and *A. niger* could be shown [94]. These findings correlate with those from another study, where the antimicrobial potential of isolated *C. acanthifolia* EO and drug decoctions (water, wine, and vinegar) was examined. Both, *C. acanthifolia* EO and the vinegar decoct, exhibited strong antimicrobial activity against several human pathogens (MIC = 0.02–0.78 µL/mL). Among them, *S. aureus* was reported to be the most sensitive strain, followed by Gram-negative bacteria such as *Proteus vulgaris*, *P. aeruginosa*, *E. coli*, and *K. pneumoniae*, as well as *C. albicans.* Compared with the aforementioned EO, the antimicrobial activity was 13-fold higher, which seems to be attributed to interactions of other (minor) constituents with carlina oxide [88]. Congruently isolated carlina oxide demonstrated strong antimicrobial activity (MIC = 15 µg/mL) against Gram-positive MRSA strains and *S. pyogenes*, alongside the fungi *C. albicans* and *C. glabrata*. Gram-negative bacteria such as *P. aeruginosa*, *K. pneumonia*, *E. coli*, and vancomycine resistant Enterococcus (VRE) were less susceptible to carlina oxide (MIC = 60 µg/mL) [97]. Apart from that, isolated carlina oxide has recently demonstrated potential as a natural food preservative, exerting promising antioxidant and in vivo antifungal activity to control infection of apples by *P. expansum* [98]. Besides antimicrobial properties, the EO from *C. acanthifolia* roots revealed a high degree of anti-inflammatory activity, comparable with that of indomethacin used as a reference drug. The observed dose-dependent gastroprotective activity was even better than that of the reference anti-ulcer drug, ranitidine. Furthermore, a considerable and dose-dependent anti-oxidative activity and high DPPH scavenging activity (IC_50_ = 13.6 µL/mL) could be observed, with carlina oxide being identified as the main antioxidant. The authors presumed that the observed anti-inflammatory and anti-ulcer properties could also be ascribed to carlina oxide [94]. The antioxidant potential of carlina oxide has strong potential as a shelf-life preservative of herbal-based insecticides [81].

Various recent studies have highlighted the promising impact of *C. acaulis* root EO as a source for botanical insecticidal products [10,81,95,97,99,100]. Insecticidal screening of eight different EOs against *Prostephanus truncatus* and *Trogoderma granarium* adults—two stored-product beetles—revealed *C. acaulis* as an effective alternative grain protectant [10]. Another study elucidated *C. acaulis* EO formulated in protein baits, to be highly toxic (LC_50_ = 1094 ppm) and aggressiveness-inhibiting in the adult medfly *Ceratitis capitata*. Overall, the EO turned out to be a good candidate for the development of eco-friendly formulations, used in medfly “attact and kill” approaches [95].

Furthermore, *C. acaulis* EO and carlina oxide are currently indicated as the most toxic botanical larvicides (LC_50_ = 1.31 μg/mL and LC_90_ = 1.83 μg/mL) against *Culex quinquefasciatus* larvae, commonly known as the southern house mosquito. Its effectiveness might be partly correlated with the AChE inhibitory properties of carlina oxide [81]. Finally, the outstanding efficacy against *Cx. quinquefasciatus* led to the recent evaluation of micro- and nanoemulsions (ME and NE) containing 0.5% of *C. acaulis* EO or carlina oxide as active ingredient. Thereby, EO-based ME was found to be the strongest larvicide, with LC_50(90)_ of 579.1 (791.3) μL/L. The application, even at the lowest LC tested (LC_16_) led to 100% insecticidal efficacy at 18 days [101].

Apart from that, *C. acaulis* EO and a highly stable EO-based NE—developed by Benelli and colleagues—exhibited high toxicity against 1st instar larvae of the European grapevine moth *Lobesia botrana* (LC_50_ = 7.30 and 9.04 µL/mL for *C. acaulis* EO and NE, respectively). However, if applied at the same concentration, the EO encapsulated in the NE was found to be more effective than pure *C. acaulis* EO. This might be related to a better interaction between the active substance and the target site, alongside a guaranteed conservation of the EO’s insecticidal activity through encapsulation, ensured dispersibility in the environment, and long-time stability [100]. Moreover, *C. acaulis* EO exerted an outstanding toxicity against (3–5 days old) housefly *Musca domestica* L. adults (LD_50_ = 2.74 and 5.96 µg/fly for males and females, respectively). Additionally, a negative impact on females’ (selective) fecundity in sublethal concentration (LD_30_) was reported for the first time. It was stated that the lipophilic nature of the EO’s main compound carlina oxide might allow penetrating the exocuticle, enabling its effects inside the insect tissues [99]. Apart from that, isolated carlina oxide demonstrated a strong and selective antiprotozoal activity against *Trypanosoma brucei brucei* (IC_50_ = 1.0 µg/mL). This effect may be attributed to its capacity to alkylate thiol groups of trypanothione enzyme through its triple bond [97].

Besides its strong potential as an ingredient of botanical insecticides, previous reports on possible cytotoxic effects of the EO [81,88,97] have recently been strengthened by indicating high dose- and time-dependent cytotoxicity on normal human fibroblasts (IC_50_ = 9.4–14.2 µg/mL after 6–48 h) [99]. Those results correlate with another study evaluating the possibility of reintroducing *C. acaulis* root into phytotherapy and reporting carlina oxide to be highly toxic in vivo and in vitro. Besides a high in vivo toxicity of LC_50_ = 10.13 µg/mL after 96 h of exposure, teratogenic and cardiodepressive effects on zebrafish embryos were also observed [5]. Contrarly, a recent conducted study reported only mild toxicity of the pure EO to human keratinocytes (IC_50_ = 115.92 ± 6.1 µg/mL) and human fibroblast (IC_50_ = 88.31 ± 1.3 µg/mL) cell lines. Also, in rats, *C. acaulis* EO appeared to be mild toxic, with LD_50_ = 1098 mg/kg. However, this toxicity was negligible when the EO was encapsulated into the ME [101].

To draw final conclusions for possible restrictions at a food level, authors encourage food safety authorities to perform a full toxicological assessment of *C. acaulis* EO and carlina oxide [5,99,101]. Current data suggest that *C. acaulis*-based extracts considered for therapeutic use should be completely deprived of carlina oxide [5].

### 2.4. Chrysopogon zizanioides

*Chrysopogon zizanioides* (L.) Roberty (CZ) (formerly *Vetiveria zizanioides* (L.) Nash) is a member of the Poaceae family and commonly known as vetiver, Khas-Khas or khus grass [102]. It is a tall, tufted, perennial, scented aromatic grass, native to India, and known since ancient times. It is widely distributed to India, Burma, Ceylon, and Bangladesh and spread from Southwest Asia to topical Africa [102,103]. Two varieties of vetiver are recognized in India: the wild growing, flowering/seeding variety in North India and the non-seeding variety cultivated in South India [104]. Vetiver has a straight stem, which is characterized by long narrow leaves, and an abundant, complex and extensive root system [103]. Contrary to other grass forms having mat-like root systems, *C. zizanioides* grows in large clumps of a heavily branched, ‘spongy’ rootstock with erect culms. The long, fibrous roots and rootles reach a depth of 2–3 m [102,105]. Based on these characteristics, the vetiver plant is highly drought-tolerant and can help to protect soil against sheet erosion. Therefore, it is widely used in agroforestry management and flood control. Moreover, vetiver has been found to be a promising aromatic grass for phytoremediation of heavy metal contaminated sites as well as for wastewater treatment, and pollution mitigation [106,107,108,109]. Apart from that, vetiver roots are widely used as raw material for curtains, mats and fans, thanks to their sweet cooling and long-lasting aroma [110].

For therapeutical purposes, vetiver root infusion has been used as a refrigerant, febrifuge, diaphoretic, stimulant, stomachic, antispasmodic, emmenagogue, astringent, blood purifier, spermatorrhoea, and strangury [111]. In folk medicine, vetiver and its root oil are further well known for their beneficial effects in the treatment of mental and emotional symptoms and their relaxing/sedative effects [112]. The EO is also claimed to possess anti-inflammatory, antiseptic, aphrodisiac, cicatrisant, tonic, and vulnerary efficacy, as well as benefits in strengthening bones, the treatment of rheumatism, gout, arthritis, muscle aches, dryness, cramps, and dry skin [110]. Since ancient times, the root oil has further been used in treatment against obstinate vomiting, colic, and flatulence, as well as a stimulant and diaphoretic [113]. Moreover, *Chrysopogon zizanioides* EO has a long history of use for its insect-repellent properties [114]. Overall, vetiver and its EO hold enormous commercial value for environmental, agricultural, food, and medical applications, as well as for perfumery and aromatherapy [115].

Vetiver EO is found to be extraordinarily complex in the number and structure of constituents as well as the odorous picture. Its fragrance can be deduced to numerous sensorially important constituents, being described as strong, warm, balsamic-woody, with sandalwood, cedarwood, ambery, and patchouli aspects and sweet-sour grapefruit- and rhubarb-like notes [116]. The key odorant, responsible for this characteristic smell—which is a mystery until today—could be shown only recenty. 2-Epi-ziza-6(13)-en-3-one was proven as the active smelling principle of vetiver oil by an eleven-step chemical synthesis, employing a novel asymmetric organocatalytic Mukaiyama-Michael addition [117]. Vetiver EO is further characterized by high solubility in alcohol, which improves its miscibility with other perfumery material. These properties make vetiver oil one of the finest and most popular raw materials in perfumery as a fixative and a fragrance ingredient. Thus, it appears in over a third of all fragrances, added to various products such as perfumes, deodorants, lotions, soaps, cosmetics, etc. [110,113,117]. The US Food and Drug Administration (FDA) classified *C. zizanioides* root EO as GRAS (generally recognized as safe) and approved the oil for use as a food and flavor additive in alcoholic beverages, chewing gum, candies, dairy, and baked food products [118].

The chemical composition of *C. zizanioides* EO has been extensively studied. To date, over 300 compounds have been isolated, mainly dominated by sesquiterpenes and their derivates such as alcohols, hydrocarbons, and ketones [116,119]. Nevertheless, many constituents still remain unidentified or poorly characterized and some controversies exist among the components identified, as reviewed by Belhassen et al. [120]. Literature data indicate that the chemical composition depends on many factors, different genotypes and geographical origins playing a key role [120]. Comparison of 21 accessions of vetiver (*V. zizanioides*, sterile, oil type) and Khus (*V. zizanioides*, fertile, non-oil type) suggested that there seems to be a significant interaction between genotypes and growing locations on oil yields, with contents ranging from 0.29% to 9.61%. The cultigen “Sunshine” was generally confirmed as delivering the highest yield (above 5.7%). However, the percent concentration varied less than the absolute yields (*g*/*g*), with khusimol (14.5–31.4%) Figure 1(15), (*E*)-isovalencenol (9.8–16.5%) Figure 1(16), β-vetivone (2.2–7.1%) Figure 1(17), and α-vetivone (3.2–5.7%) (Figure 1/18) as the major compounds among all of the cultivars. By comparison, commercial oils differed in composition, underlining the impact of extraction differences, genetic variations, unknown edaphic factors, and/or oil adulterations [121]. Comparative analysis of commercial EOs obtained from *C. zizanioides* root samples from nine different countries (Brazil, China, Haiti, India, Java, Madagascar, Mexico, Reunion, and Salvador) enabled the identification of 114 constituents, accounting for 73.7–89.4% of the total oils. Whatever the origin of the plant, no significant qualitative differences could be observed, if derived from the same cultivar (Sunshine). Khusimol (3.4–13.7%), β-vetispirene (1.6–4.5%), vetiselinenol (1.3–7.8%), and α-vetivone (2.5–6.3%) were the main constituents throughout the world [119].

South Indian vetiver oils from four different locations, were characterized by nootkatol + khusimol (16.1–19.2%), isonootkatol + isovalencenol (5.6–6.9%), vetiselinenol + (*E*)-opposita-4(15),7(11)-dien-12-ol (3.7–5.9%), 13-nor-trans-eudesma-4(15),7-dien-11-one + amorph-4-en-10-ol (5.0–6.4%), β-vetivenene (0.9–9.4%), and δ-selinene + β-vetispirene (3.9–6.1%). It was stated that the characteristic vetiver odor was related to khusimene, δ-selinene, β-vetivenene, cyclocopacamphan12-ol (epimers A and B), vetiselinenol, khusimol, isovalencenol, khusimone, α-vetivone, and β-vetivone [104]. Claimed as the ‘typical’ vetiver oil, South Indian vetiver oil is dextrorotatory in nature, while the North Indian vetiver oil is laevorotatory and devoid of α- and β-vetivone. By contrast, the latter is rich of antipodal sesquiterpenes of the cadinene type [122].

Steam distillation of vetiver roots from the ‘Sri Lanka’ ecotype from Northeast of Thailand yielded 0.3–1.0% *v*/*w* of viscous light-brown oil with a balsamic earthy and sweet woody odor. It was characterized by khusimol (12.7%), longipinene (4.2%), valerenol (3.9%), epizizanal (3.3%) as the main constituents, followed by α-vetivone (2.0%), and β-vetivone (1.6%) [113]. Phytochemical analysis of vetiver EO from Comoros—obtained by hydrodistillation (1% *v/w* yield)—showed the presence of 40 constituents, representing 99.98% of the oil. Khusimol (25.6%) was found to be the main constituent besides bicyclo-vetivenol (11.5%), α-vetivone (7.8%), epi-α-cadinol (6.0%), and nootaktone (5.3%), followed by khusinol acetate (3.8%), nootkatol (3.5%), 1,10-epi-cubenol (3.1%), and khusinol (3.0%) [123]. Regarding commercial vetiver EO from Thailand, α-vetivone (9.0%), vetivenic acid (7.7%), β-vetivenene (4.5%), and khusimol (3.9%) were found to be the principal components [124]. The analysis of vetiver root EO from South Asia showed the presence of 27 constituents, with cedr-8-en-13-op (14.5%), α-gurjunene (9.8%), α-amorphene (8.0%), 3,8-dimethyl-4-(1-methyl-ethylidene)-2,4,6,7,8,8a-hexahydro-(7.7%), β-guaiene (6.3%), and solavetivone (5.2%) as the major compounds [125]. The commercial oil from Taiwan with 25 identified compounds was similar in its complexity and main constituents with cedr-8-en-13-ol (12.4%), and α-amorphene (7.8%). Other dominating ingredients were β-vatirenene (5.9%) and α -gurjunene (5.9%) [126]. Similarly, cedr-8-en-13-ol (12.7%) and β-vatirenene (7.3%) were identified as the main constituents among 37 identified components from Indonesian commercial vetiver EO [108].

Massardo et al. reported that—particularly during early root growth—vetiver EO production was closely related to the metabolism of the roots, which in turn was affected by changes in environmental temperatures: reduced temperatures resulted in reduced metabolic plant activities, which consecutively slowed down EO production. Thus, changes in the environmental temperatures seem to affect symbiotic bacteria. Those root-associated microorganisms are believed to play a crucial role in the biotransformation of plant-derived terpenoids to produce the complex vetiver EO, based on a collaborative effort [105]. Finally, these results may open the possibility to manipulate the molecular structure of the vetiver oil, either in vivo or in vitro [127,128]. This hypothesis has been strengthened by previous publications reporting that vetiver cleansed by bacteria and fungi produced only trace amounts (0.02%) of oil and revealed a strikingly different composition compared with the oils from non-cleansed vetiver plants (0.35%), grown in the same kind of soil and under the same conditions [129].

However, studies on the phytoremediation properties of *C. zizanioides* revealed that vetiver EO production (0.4–1.3%) and composition (47–143 compounds) furthermore significantly increased by the uptake of heavy metals Pb [130] and Cd (101.56% total content increase by 100 ppm Cd-HM treatment). By contrast, oil production decreased with a higher water content in the plant [131]. Recent obtained results further elucidated quantity and quality variations of vetiver EO according to different red mud treatments (0, 5, 10, and 15% *w*/*w*). With increasing treatment, contents of rosifoliol, α-muurolol, farnesol, γ-costol, isovalencene, and vetivone increased, while selina-6-en-ol, cadin-4-en-10-ol, vetiselinenol, and aristolone decreased. Results suggest the utilization of 5% and 10% red mud in sludge-amended soil for improved quantity and quality of vetiver EO, without any metal contamination coupled with enhanced phytoremediation potential of vetiver [132].

Additionally, EO recoveries also seemed to depend on the storage and preparation method of vetiver roots prior to distillation process. The yield progressively decreased with the period of storage, while cutting fresh roots to lengths of 2.5–5 cm increased recovery [110]. Finally, it could be shown that the yield and composition of vetiver EO strongly depended on the methods of cultivation. Accordingly, total oil recoveries in three different cultivation systems (normal soil, normal soil with added microbes, and semi-hydroponically) varied between 0.006% and 0.27%. The highest yield was obtained from cultivation in normal soil with added microbes [133].

The potential role of *C. zizanioides* in traditional medicine for the treatment of various diseases has prompted several phytochemical studies elucidating multi-functional biological activities of its EO [134]. According to the literature, vetiver EO and its constituents revealed antimicrobial activities against various pathogens, mainly Gram-positive strains, particularly *S. aureus*, either susceptible (MSSA) or resistant to methicillin (MRSA) [108,135,136].

Regarding the important role claimed of vetiver EO as an ingredient in cosmetics, Burger and co-workers evaluated its preservative potential by assessing bacteriostatic effectivity against 20 bacterial strains. *S. aureus* (both susceptible and resistant to methicillin), *Corynebacterium striatum* and two *Bacillus* strains—with MICs comprised between 500 and 2000 µg/mL—were reported as the most susceptible microbes [136]. Potent antimycobacterial activity against drug-resistant strains of *Mycobacterium smegmatis* (MIC = 31.25–62.5 µg/mL) was reported for vetiver EO, its fractions and the isolated compounds 5,10-pentadecadiyn-1-ol, α-curcumene, hydroxy junipene, (+)-cycloisosativene, valencine, and selino 3,7(11)-diene [137]. Ramirez-Rueda et al. were first to report a bioguided analysis of *C. zizanioides* EO against multidrug-resistant *S. aureus* and *E. faecalis* based on TLC bioautography. They proposed that the observed antibacterial activity in their tested EO was mainly linked to cedr-8-en-13-ol, aside from 6-isopropenyl 4,8a-dimethyl-1,2,3,5,6,7,8,8a-octahydro-naphthalen-2-ol, δ-selinene, γ-gurjunenepoxide-(2), and solavetivone [108].

In addition to *E. faecalis* (MIC = 31.25 μg/mL), antibacterial activity against *Enterobacter cloacae*, *E. coli*, and *P. vulgaris* (MICs = 15.63, 15.63 and 15.63 μg/mL, respectively) has been reported [138]. Moreover, only recently, another research group indicated potent antimicrobial activity of *C. zizanioides* EO against *S. aureus* (MIC = 50 ± 0 μg/mL), followed by *P. mirabilis* (MIC = 75 ± 5 μg/mL). Furthermore, a synergistic effect with *Ocimum basilicum* leaf EO on various microorganisms has been reported. An increased synergistic activity of this EO mixture as vapor treatment could also be observed against post-harvest phytopathogen *P. notatum* in Jackfruit, thus showing potential as a preservative [125].

*Rhizoctonia solani*—another phytopathogenic fungus—was found to be sensitive against both types of Indian vetiver oils in a dose-dependent manner, with the South Indian type being slightly more effective compared with the North Indian type [139]. Furthermore, preliminary in vitro studies indicated that vetiver root EO exerted strong activity against the wood rot fungi *Gloeophyllum trabeum*, *Poria placenta*, *Coniophora puteana*, and *Coriolus versicolor*, thus showing potential in the conservation and protection of softwood [123]. Regarding its antifungal properties, *C. zizanioides* oil also exerted moderate inhibitory activity on *C. albicans* (MICs = 200–800 µg/mL) and strong activity against *C. glabrata* (MICs = 100–400 µg/mL) [136]. In agreement with its traditional use as an insect repellent, several publications indicated that vetiver oil and some of its components exerted repelling actions against a variety of insect species. Preliminary studies on vetiver EO with vetiveryl acetate, vetiverol, vitivone, and terpenes as its main chemical components showed a promising potential mosquito repellent and/or irritant activity against *Anopheles minimus*, a malaria vector in Thailand [140]. Khater and co-workers investigated the insecticidal effectiveness and growth inhibition potential of vetiver, cinnamon, and lavender EO against second (L2) and third larval (L3) stages of the sheep blowfly *Lucillia sericata*, an economically important ectoparasite of humans, as well as livestock, pets, and wildlife species. The results indicated vetiver oil (5%) as the second most effective biopesticide, after cinnamon oil, exerting high larval mortality on *L. sericata* (L2), 24 h post treatment (84.44%) and adverse effects on the subsequent developments [141]. However, the answer of *Halyomorpha halys* stink bugs to vetiver EO was ambiguous. At 25%, it elicited both a strong repellency in the active during summer physiological–behavioral phase, and a strong attraction in exiting overwintering bugs [142].

Additionally, the dose-dependent, high nematicidal efficacy of vetiver EO against the widely-spread root-knot nematode *Meloidogyne incognita* could be shown. Egg hatch inhibition as well as mortality of second-stage juveniles (J2s) were reported [143]. Vetiver EO was found to be a potential alternative termiticide, exerting strong repellency and antifeedant activity against the Formosan subterranean termites, *Coptotermes formosanus*, with nootkatone being one of the major chemicals in the EO responsible for the termiticidal activity. At a concentration of 5 µg/g sand, termite’s tunneling was reduced, and entirely inhibited together with paper consumption at concentrations higher than 25 µg/g sand [134]. Moreover, α-vetivone, β-vetivone, khusimone, zizanal, epizizanal, and (+)-(1S, 10R)-1, 10-dimethyl bicyclo [0,4,4]-dec-6-en-3-one are known to possess repellent properties against arthropods [144]. Finally, significant potential acaricidal activity of *C. zizanioides* EO on two tick species, *Amblyomma cajennense* and *Rhipicephalus microplus*, was also described by reducing the egg number, egg hatch, and causing mortality [145].

Modern scientific studies have confirmed that vetiver EO possesses potential sedative/relaxing and anxiolytic effects upon inhalation [113,146]. Furthermore, oral administration of vetiver EO (2 mL/kg) induced a significant sedative and hypnotic effect in mice, probably by facilitating the GABAergic pathway. The effects were similar to those observed with 5 mg/kg of the standard sedative-hypnotic drug diazepam in terms of latency and total sleep duration. Furthermore, a potentiated effect of phenobarbital induced sleep has been reported. Altogether, vetiver EO may provide comparable therapeutic efficacy with diazepam in insomnia [111]. Moreover, it has been reported that breathing the volatile components emitted from the EO of *C. zizanioides* reduces blood volume in the frontal lobe of brain, underlining the potential sedative effects on brain activity [147]. Besides that, vetiver balm could reduce plasma cortisol hormone level in prolonged swim-induced stress rats in a concentration-dependent manner, stating that EO titration may have a stronger effect. Thus, vetiver balms showed a beneficial effect as an anti-stress treatment [112].

On the contrary, vetiver EO inhalation has also been suggested to be beneficial for learning and memory processing [124]. Inhalation of volatile compounds emitted from the cut roots of *C. zizanioides* increased focus in humans during a visual display terminal task. Participants under low-dose conditions (0.25 μg) showed faster reaction times and a stimulated sympathic nerve activity [148]. Inhalation of vetiver EO with β-vetivenene, khusimol, vetivenic acid, and α-vetivone as its main constituents also showed remarkable refreshing effects by increasing total waking and reducing slow-wave sleep time. Hence, it is also considered as a stimulant to improve alertness and task performance [124].

In the context of its traditional use, vetiver EO has been evaluated for its anticonvulsant properties. At a dose of 300 mg/kg in maximal electroshock (MES)-induced seizures in mice, vetiver EO showed 100% protection (1/1, 4 h), indicating the compound’s ability to prevent seizure spread. At this highest administered dose (300 mg/kg), no neurotoxicity was observed. Vetiver EO did not show protection in subcutaneous pentylenetetrazole (PTZ)-induced seizures [149]. However, in a subsequent study, 250 and 500 mg/kg vetiver EO were found to have equal anticonvulsant efficacy as sodium valproate in a PTZ model [150]. In the Siddha system of medicine, *C. zizanioides* root has been used to treat hypertension. The vasorelaxation effect of its EO has been recently assessed. Vetiver EO nano emulsion significantly induced relaxation (E_max_ 77.1 ± 4.87%) in phenylephrine (1 µM) precontracted aortic rings through a muscarinic pathway as well acts as a calcium channel blocker [151].

In several studies, vetiver EO has also been found to be an excellent natural antioxidant [125,134]. Pure vetiver EO revealed a powerful anti-oxidative and DPPH free radical scavenging activity compared with standard antioxidants such as butylated hydroxytoluene (BHT) and α-tocopherol [134,152]. The authors indicated that this activity might be related to its β-vetivenene, β-vetivone, and α-vetivone content, as they exerted strong antioxidant activities when tested individually [152]. Associated with this strong anti-oxidative activity, vetiver EO further demonstrated an impact as a chemopreventive drug, reducing cisplatin-induced nephrotoxicity, genotoxicity, and myelosuppression in mice when administered in combination with cisplatin [153].

Apart from this anti-oxidative properties, vetiver EO has also been found to exert anti-inflammatory activity on LPS-stimulated murine macrophage cells—including NO production and cell apoptosis—by modulating the expression of inflammatory-related enzymes (HO-1, iNOS, and COX-2) and cytokines (TNF-α, IL-1β, and IFN-β). It could be shown that the anti-inflammatory activity of vetiver EO related to its antioxidant ability of decreasing LPS-induced superoxide anion production and malondialdehyde (MDA) levels [126].

Another result of its potent anti-oxidative function might be the ability of vetiver EO to reduce the α-MSH-stimulated melanogenesis in murine B16 melanoma cells by dowregulation of tyrosinase activity and expression in a dose-dependent manner. Consequently, vetiver EO has potential as an ingredient in hypopigmentation drugs, foods, and cosmetics [154]. By contrast, Burger et al. demonstrated only weak anti-oxidative activity, concluding that a larger set of vetiver EOs should be evaluated using several antioxidant tests (DPPH, ORAC, etc.) to solve this contradiction. The authors concluded that vetiver oil does not seem to display any interesting in vitro anti-aging activity. No inhibitory activity of collagenase or hyaluronidase was observed and only a weak anti-elastasic activity was noted [136].

However, Han et al. were the first to investigate the biological activity of vetiver oil in dermal fibroblasts, indicating strong anti-proliferative activity as well as inhibitory effects on collagen III production, an important molecule for skin and tissue remodeling processes. However, probably due to different mechanisms of action, no inhibitory effect on inflammatory responses in human dermal fibroblasts could be found. Apart from that, diverse effects on regulating human genes were observed, influencing cholesterol synthesis and metabolism. Although further studies on how *C. zizanioides* EO influences these genes are necessary, the researchers concluded that vetiver EO may be a potential candidate for skincare and obesity [114]. Finally, the hypolipidemic effect of *C. zizanioides* EO in high fat diet-induced hyperlipidemia rats was evaluated, elucidating significant hypolipidemic activity, which was suggested to be associated with its underlying antioxidant properties [155].

Investigations into the anticarcinogenic properties from vetiver EO elucidated growth inhibition at 100 ppm in cancer cell lines, with up to 89% of SiHa cervical cells, 88% of CaSki cervical cells and 89% of MCF-7 breast cancer cells [134]. However, although vetiver acetate was found to be significantly genotoxic and cytotoxic in human lymphocytes at high concentrations, vetiver oil is considered safe for human consumption at low concentrations [115].

### 2.5. Coleus forskohlii

*Coleus forskohlii* Briq. (syn. *Plectranthus barbatus* Andr.)—known as Coleus—is an aromatic perennial herb and one of the most important *Plectranthus* species. The plant is a member of the Lamiaceae family, and it is considered to originate in the Indian sub-continent. It grows under tropical to temperate areas in India, Burma, Thailand, Nepal, Pakistan, Sri Lanka, East Africa, and Brazil [156]. *Coleus forskohlii* has an erect fleshy, 30–90 cm high stem with broadly ovate or cordate aromatic leaves and flowers in racemose of 6–10-flowered cymes [157]. The roots of the rootstock are fasciculate, thick, and succulent, tapering with few rootles. They are described to be pale brown in color, slightly bitter to pungent in taste and characterized by a pleasing aromatic odor [157,158,159]. With the entire plant being aromatic, leaves and tubers significantly differ in their odor [159]. Thus, the EO is known for its potential uses in the food flavoring industry and as an antimicrobial agent [160].

Coleus is very important in ethno-medicinal uses to treat various diseases [161]. The rootstock of the plant has been used since ancient times in Ayurveda and other systems of medicine for several ailments such as heart and lung conditions, asthma, digestive disorder, liver complaints, insomnia, muscle spasm, convulsion, and skin diseases [156]. Clinical studies of the plant and its main constituents support these traditional uses, ascribing most of the pharmacological actions to forskolin (coleonol), a labdane diterpenoid found exclusively in the tuberous roots of *C. forskohlii*. Its unique direct adenylyl-cyclase activation properties in diverse tissues endows forskolin with significant proven therapeutic benefits against various ailments, such as cystic fibrosis, asthma, obesity, cardiovascular diseases, cancer, diabetes, glaucoma, and liver fibrosis [162,163].

Nevertheless, besides forskolin as the leading principle, EOs have been found to be the main components in the tuberous roots of *C. forskohlii* [163]. Coleus oil is characterized by a very attractive and delicate odor with a spicy note [164], and it can be obtained as a valuable byproduct of forskolin-rich extract production, without detrimental effects on the yield of forskolin [165].

However, although *C. forskohlii* is a highly aromatic herb, the chemistry and biological properties of its EO remain poorly known. Only a few phytochemical investigations have been conducted into the EO composition from *C. forskohlii*, indicating a tremendous variability in the chemical composition between different vegetative organs [166,167], as well as accordingly to different genotypes, plant origins, and grown altitudes [164,168]. Those variations may also be caused by pedoclimatic conditions and agricultural practices adopted [166]. However, the presence of 3-decanone Figure 1(19), bornyl acetate Figure 1(20), sesquiterpene hydrocarbons, and sesquiterpene alcohols in major concentrations gives the oil a unique combination of an attractive spicy and long-lasting odor [164].

Misra et al. analyzed the variations in the chemical composition of the EO obtained from different genotypes of wild growing *C. forskohlii* roots collected from different parts of India. The hydrodistillation of the samples revealed a viscous, dark brown-colored oil, with yields ranging from 0.03% up to 0.19% (fresh wt basis). Within over 40 recovered compounds, sesquiterpene hydrocarbons (39.2–64.8%) had the strongest contribution to the EO content, followed by sesquiterpene alcohols (16.9–37.3%), monoterpenoids (8.7–38.4%), and diterpenoids (2.2–6.8%). The main compounds identified from the EOs derived from these genotypes were bornyl acetate (15.0%), β-sesquiphellandrene (13.2%), and γ-eudesmol (l2.5%). Other dominating components were 3-decanone (7.0%) and α-gurjunene (5.0%), aside an unidentified sesquiterpene hydrocarbon (7.5%) and sesquiterpene alcohol (6.3%) [164]. On the contrary, steam distillation of *C. forskohlii* roots grown in Brazil yielded higher amounts of total oil (0.45%), although they strongly differed in the presence and concentration of components. (*Z*)-β-Ocimene (37.6%) and α-santalene (8.1%) were identified as its leading compounds, aside from β-caryophyllene (6.8%), bornyl acetate (6.2%), sabinene (6.0%), (*E*,*E*)-farnesol (5.6%), and α-pinene (4.7%) [167]. The rare aromatic diterpene abietatriene (0.7%) was reported for the first time [164,167]. However, the EO obtained by steam distillation from roots collected in north Italy revealed β-caryophyllene (15.1%) almost twice in concentration. Other main constituents were β-phellandrene (10.6%), β-gurjunene (10.6%), as well as humulene (6.3%) [166]. Analysis of another Indian *C. forskohlii* root sample led to the identification of 45 volatile compounds. The EO was dominated by decanal (18.4%) Figure 1(21) and bornyl acetate (16.9%), alongside decyl acetate (9.2%) Figure 1(22), cetene (8.9%), and 1-decanol (5.5%) as further dominating constituents. Moreover, α-pinene (4.4%), n-decanoic acid (3.9%), camphene (3.7%), β-pinene (3.6%), α-humulene (3.4%), borneol (2.6%), 1,8-cineole (1.7%), and α-ylangen (1.6%) were listed as the main ingredients [169]. Similarly, bornyl acetate (13.0%) and n-decyl acetate (6.1%) were registered as the major components in the Indian *C. forskohlii* root EO assessed by Takshak et al. Decanal (30.7%)—as the principal component—was found in significantly higher amounts than previously reported. The hydrodistilled EO was further characterized by *E*,*E*,*Z*-1,3,12-nonadecatriene-5,14-diol (4.4%), β-sesquiphellandrene (3.8%), β-pinene (3.4%), guaia-1–10, (11)-diene (3.4%), and 8-propoxycedrane (2.7%). The same research group was the first to investigate the impact of supplemental UV-B (s-UVB) treatment on *C. forskohlii* plant extracts and EO composition and content. It could be shown that the EO composition considerably altered under stress condition, while the content was found to be reduced (by ∼7%) in the oil. Pharmacologically-relevant constituents such as α-pinene, d-camphene, decanal, and sclareol increased in the EO (6.2–841.7%), while others such as β-pinene, myrcene, γ-terpinene, nonanal, α-cis-bergamotene, β-transbergamotene, and intermedeol decreased in concentration (33.3–95.2%). Furthermore, some compounds were detected in the oil obtained from s-UV-B-treated plants, while others were not detectable [170]. The EO of *C. forskohlii* roots from India—extracted and purified by a proprietary patent process—was characterized by bornyl acetate (15.0%), 3-decanone (7.0%), and an azulene derivative (sesquiterpene) (7.5%) as its principal components, as well as α-pinene (2.0%) and β-pinene (1.5%). Other constituents identified included α-humulene, α-gurjunene, and α-selinene [165]. However, another root EO of *C. forskohlii* obtained by SFE- extraction method was dominated by α-pinene, endo-borneol, bornyl acetate, 1-hexyl-2-nitrocyclohexane, and phytol. In total, 16 constituents were identified [171].

The EO was further investigated for its antimicrobial activity, demonstrating its potential in the treatment of microbial infections. The tested oil, obtained by a proprietary patent process, showed remarkable inhibitory action against skin pathogens such as *Propionibacterium acnes, S. aureus*, *S. epidermidis*, and good activity against *E. coli*. Moreover, the same EO was found to be effective against *Streptococcus mutans,* a leading organism in dental caries, as well as *C. albicans* [165]. Due to its antimicrobial activities, the composition of investigated *C. forskohlii* oil was found to be useful in various topical preparations. These include skin care formulations for the treatment of skin infections such as acne, as well as oral and topical preparations for candidal infections. Finally, no skin irritation, erythrema or edema of skin on rabbits were observed, underlining the potential of this EO composition in the treatment of microbial infections [165].

Only recently, the root EO of *C. forskohlii* was furthermore evaluated for its potential application in food industries and clinical settings. Thereby, promising antibacterial activity was found against *L. monocytogenes*, *S. typhimurium*, and *E. coli* (at EO concentrations of 3.12% *v*/*v*, 6.25% *v*/*v*, 6.25% *v*/*v*, respectively). Additionally, quorum sensing inhibition in *Chromobacterium violaceum* and *P. aeruginosa* was reported. In all the tested pathogens a significant dose-dependent inhibition for biofilm formation and motility could be observed [171].

Investigations on the antiradical activity of different vegetative organs from *C. forskohlii* revealed good DPPH and ABTS quenching activities from the roots (stems > roots > leaves), with an IC_50_ of 0.52 mg/mL (DPPH test) and 0.43 mg/mL (ABTS test), respectively. According to the authors, the antiradical activity of the root EO could be ascribed to a synergistic effect between several terpenic structures. Furthermore, a metal-chelating activity on ferrous ions (Fe^2+^) is reported at a concentration of 60 µg/mL with a value of 19% [166].

### 2.6. Inula helenium

*Inula helenium* L.—also known as elecampane—is a widely occurring perennial plant species from the Asteraceae family [172]. It is native to South and East Europe with a widespread distribution in Asia, Central Europe, and North America. The stem of the herb attains a height of up to 2.5 m, with large and irregular toothed leaves. Flower baskets have very narrow ray florets and many small tubular florets [13]. In Eastern Europe, elecampane has been traditionally used for the treatment of asthma, bronchitis, and whooping cough, as well as digestive disorders, urinary infections, and skin disorders [33]. In traditional Chinese medicine (TCM), the roots have been used as a substitute for medicinal herbs to relieve pain associated with abdominal distention and indigestion with anorexia, nausea, and vomiting [173].

*Inula helenium* roots mainly comprise the polysaccharide inulin (up to 44%) [13], aside sesquiterpene lactones, thymol derivates, phenolic acids, and flavonoids [174]. Elecampane EO (1–5% in the roots) is described as woody, balsamic, and earthy in its odor [175], being solid at room temperature. It contains a mixture of white crystals of sesquiterpenoid lactones and a liquid yellow phase attributed to alantol [176]. However, due to its sensitizing potential [177], the IFRA recommends that elecampane oil should not be used as a fragrance ingredient [22]. As one of the richest sources of sesquiterpenoid lactones [178], this fraction represents at least 88% of the total EO [176]. Minor constituents, such as monoterpenoids, only occur in small amounts. Nevertheless, the main constituents of *I. helenium* EO differ according to the origin of the plant and extraction method. Thus, the number of constituents identified in each root sample varied from 17 to 70 compounds [176,179].

Hernandez-Ochoa et al. compared the composition of elecampane EOs from South France, obtained by hydrodistillation and co-hydrodistillation, using 10 mL ethyl heptanoate as co-solvent. Both, the oil complexity (20–70 constituents) and content of the principal components—identified as alantolactone (ALT) (49.5–56.6%) Figure 1(23) and isoalantolactone (IAL) (32.2–37.3%) Figure 1(24)—were significantly higher in the EO obtained by hydrodistillation. Nevertheless, 10% of minor volatiles such as α-pinene, camphene, β-pinene, ethyl hexanoate, camphor, eremophilene, calarene, *trans*-acorenone, α-amorphene, α-asarone, and α-selinene were solely identified in the oil obtained from co-hydrodistillation [179]. Similarly, comparative studies on the EO composition of oils obtained by hydrodistillation (1.0% yield) and SFE (1.7% yield) in Italy revealed ALT (42.3–51.3%) and IAL (35.4–36.9%) as the main constituents in both oils. Additionally, β-elemene (1.5–1.9%) Figure 1(25) and valencene (1.0–1.5%) were identified as the most abundant components. While the amount of ALT and IAL were higher in the hydrodistilled EO, no significant difference to SFE-oil was observed in the concentration and presence of minor constituents [178]. Moreover, in the less complex EO (17 identified components) obtained by steam distillation from roots collected in Central Europe, ALT (52.4%), IAL (33.0%), eudesma-5,7(1 1)-diene-8 ß-12 olide (1.3%), and β-elemene (1.2%) were registered as the major components [176]. Hydrodistillation of elecampane roots from Serbia presented a yield of 1.4% (*w*/*w*). Phytochemical analysis identified 91.5% of the total constituents, revealing the presence of 45 different compounds. Among them, ALT (55.8%), and IAL (26.3%) were detected as the major components, similar to the aforementioned EOs, although the concentration of IAL was found to be notably lower. Interestingly, diplophyllin (5.1%) Figure 1(26) has been reported for the first time from *I. helenium* roots [33]. In another hydrodistilled *Inula helenium* EO from Serbia, less complex in its composition, 21 constituents were identified (93.4% of the total oil). Compared with the previously-reported EOs, ALT occurred with 65.8% of total oil in significantly higher amounts. On the other hand, the content of IAL (25.5%) was slightly lower [172].

Despite this large diversity in its chemical composition, phytochemical analysis clearly indicated ALT and IAL as the main bioactive compounds in the EOs, exhibiting a wide spectrum of biological activities, including antimicrobial, anti-inflammatory, and anti-cancer properties, with no significant toxicity [180]. The α, β-methylene lactone Figure 1(27) functionality—a significant characteristic of most sesquiterpene lactones—and two eudesmane core olefinic bonds in the sesquiterpene lactones seem to be essential structural requirements for their broad spectrum of activities [33,173,181]. These activities might be associated with the Michael acceptor property of this moiety, allowing the formulation of covalent adducts with nucleophilic residues on diverse biomolecules. However, the α-methylene-γ-butyrolactone functionality also seems to be responsible for the induction of several versatile skin reactions, including allergic contact dermatitis and chronic actinic dermatitis [182].

Thus, those compounds might also be responsible for the observed effect of fragrance inhalation of *I. helenium* EO (100%) on brain function. Although further studies are necessary, it seems that brain function becomes highly affected by increasing alertness and concentration. Therefore, elecampane oil inhalation may have the potential to treat psychophysiological disorders [175].

Apart from that, several studies have indicated that *I. helenium* EO and its principal constituents revealed a broad antimicrobial spectrum against various human, as well as phytopathogens, along with yeast and mold cultures [176,178]. As expected, Gram-positive strains have been found to be more sensitive to elecampane oil than Gram-negative strains [33,178]. Furthermore, stronger antimicrobial activity of hydrodistilled elecampane oils compared with SFEoils has been reported, which may be attributed to the higher content of ALT and IAL in the tested samples [178]. In particular, *I. helenium* EO revealed a potent but not fast antistaphylococcal activity (MIC = 0.013 mg/mL), with obvious membrane-damaging effects in the way of increased membrane permeability (phosphates and nucleic acid leakage), followed by lysis of the exposed cells. It was stated that ALT, IAL, and diplophyllin might be the carriers of the observed activity [33]. However, it was found that IAL did not exert noteworthy *S. aureus* toxicity as per MIC evaluation in vitro (MIC > 1024 µg/mL), but it could significantly reduce the pathogenicity of *S. aureus* in pneumonia at a very low concentration. IAL suppressed the production of α-toxin by *S. aureus* in vitro and protected mice from *S. aureus* pneumonia in vivo [183]. Furthermore, it could recently be shown that IAL synergistically increased the antimicrobial effects of penicillin G against *S. aureus* by inactivating β-lactamase. Thus, a therapy of combined IAL and penicillin G demonstrated equal effects compared with sulbactam with penicillin G [184]. These findings suggested that IAL—in combination with β-lactam antibiotics—may be a promising drug in the treatment of *S. aureus* pneumonia [183,184]. Furthermore, it could be shown that low micromolecular concentrations (1–20 µM) of ALT intensified the uptake of *S. aureus* and thus stimulated immune functions of macrophages [185]. However, IAL has also recently been found to exert strong synergistic effects with carbapenems (polymyxin B or colistin) in the treatment of mcr-1-positive Enterobacteriaceae such as *K. pneumoniae* and *E. coli* in vivo and in vitro [186].

In terms of antifungal activity, *I. helenium* EO has demonstrated strong potential as a controlling agent for various *Candida* strains by exhibiting significant anticandidal activity [172,176,178]. Furthermore, a potent synergistic effect of *I. helenium* EO against clinical *Candida* spp. isolates in combination with the antifungal agent nystatin was observed (MICs = 0.009–0.312 mg/mL). Apart from that, the EO revealed strong anti-virulence effects (anti-biofilm, germtube reducing, and phospholipase-inhibitory activity) on these human pathogens [172]. The inhibition of the virulence factors of *C. albicans* by ALT was revealed just recently for the first time, and 18–72 µg/mL of ALT could exert disruptive effects on preformed biofilms, underlining the potential of these compounds. It is stated that the antifungal effects of ALT against *C. albicans* and several other *Candida* species may involve increased cell membrane permeability and ROS production [187]. Another recent study confirmed the antifungal properties of elecampane EO by indicating considerable inhibitory effects (MICs = 50–100 μg/mL) against *C. albicans*, *C. glabrata*, *C. kruzey*, *C. parapsilosis*, *S. cerevisiae*, and *A. niger*. Among the tested fungi, the last two mentioned revealed to be the most sensitive strains [188].

In another study, IAL exerted absolute toxicity (500 μg/mL) against the soil-borne phytopathogenic fungi *Gaeumannomyces graminis var. tritici*, *R. cerealis*, and *Phytophthora capsici* as well as a weaker antibacterial activity against several other microorganisms [189]. Furthermore, isolated ALT, IAL, and 11,13-dihydroisoalantolaactone demonstrated inhibition of *M. tuberculosis* at 32 μg/mL [190]. Additionally, ALT showed a strong anti-HSV-1 impact, providing evidence for use of *Inulae radix* in the treatment of HSV-1 infection [191].

Apart from its antimicrobial properties, isolated ALT and IAL were both found to possess strong larvicidal activity against the dengue vector mosquito *Aedes albopictus* (LC_50_ = 2.7 mg/mL and 11.9 mg/mL, respectively) and the common midge *Paratanytarsus grimmii* (LC_50_ = 5.1 mg/mL and 4.1 mg/mL, respectively) [173]. However, entry-route differences might be the reason for stronger larvicidal activity of IAL against the yellow fever mosquito *Ae. aegypti* compared with ALT, as, by contrast, ALT was more efficient against adult individuals [192].

ALT and IAL have also been described as potential ailments for anti-inflammatory associated diseases by exerting immunomodulatory and anti-inflammatory effects via multiple pathways [185,193,194]. ALT increased phagocytosis, and simultaneously modulated cytokines production due to p65 NF-kB suppression [185]. Furthermore, ALT (10 µM) suppressed inducible nitric oxide (iNOS) and cyclooxygenase 2 (COX-2) expression by downregulating NF-κB and MAPK pathways in LPS-activated RAW 264.7 cells and peritoneal macrophages [195]. ALT may have also potential as an adjunctive therapeutic to treat rheumatoid arthritis by inhibiting Th17 cell differentiation through modulation of STAT3 signaling. At 50 mg/kg, ALT attenuated high arthritis scores, infiltrating inflammatory cells, synovial hyperplasia, bone erosion and levels of the proinflammatory cytokines TNF-a, IL-6, and IL-17A in a collagen-induced arthritis mouse model. [196]. Apart from that, recent observations indicated a therapeutic effect of ALT in a murine multiple sclerosis model, justifying further investigations [197].

Moreover, ALT and IAL were reported to exert neuroprotective effects and prevent ROS-mediated scopolamine-cognitive impairment through activation of Nrf2 signaling pathway and inhibiting AChE in mice. Thus, these compounds might have potential as therapeutic agents for reactive oxygen species-related neurodegenerative diseases [198]. ALT has also been found to possess strong neuro-protective potential in the clinical treatment of traumatic brain injury in rats by exerting anti-inflammatory, anti-oxidative, and anti-apoptosis pathways [193]. Furthermore, ALT has recently been shown to inhibit leucocyte adhesion to respiratory epithelial cells, strengthening the traditional use of *I. helenium* in respiratory diseases. The observed inhibitive effect on pro-inflammatory cytokines (IL-8, TNF-α, and IL-1β) and consequently β-integrin and intercellular adhesion molecule 1 (ICAM-1) was comparable with budesonide (50 µM) [199]. Apart from that, recent observations indicated a therapeutic effect of ALT in a murine multiple sclerosis model, justifying further investigations [197]. Another study investigated the function and mechanism of ALT treatment on COPD pathological process. Thereby, protective effects of ALT against inflammation, apoptosis and oxidative stress in cigarette smoke-induced human bronchial epithelial cells through activation of Nrf2/HO-1 and inhibition of the NF-κB pathways could be shown [200].

Due to its anti-inflammatory activity, IAL (20 mg/kg, i.p.) has shown potential as a therapeutic agent for the treatment of acute lung injury by significantly inhibiting pulmonary pathological changes, neutrophil infiltration, pulmonary permeability, and pro-inflammatory cytokine expression [194]. Furthermore, IAL has revealed promising potential for the treatment of osteoporosis and other metabolic bone diseases. The compound suppressed receptor activator of NF-κB ligand (RANKL)-induced osteoclastogenesis and function in a dose-dependent manner (0, 0.5, 1, 2 μM), without affecting the osteoblast differentiation [201].

Apart from IAL [202], recently particularly ALT has been extensively studied for its cytotoxic and anti-proliferative activity in a variety of human cancer cell lines, including the colon [203], lung [204], breast [205], gastric [206], pancreatic cancer [207], leukemia [208], and glioblastoma multiforme [209]. The anti-tumor properties of ALT occurred through a wide range of overlapping molecular pathways, interacting with each other [180,207]. ALT inhibited tumor cell proliferation and exerted migration suppression and apoptosis-promoting effects through increased ROS generation [203,206], glutathione (GSH) depletion [209], and inhibition of STAT3 expression [204,207]. Furthermore, ALT regulated p38 MAPK, NF-κB, and Nrf2 signaling pathways [205]. The inhibitive effect of ALT treatment (20 μM) on TrxR1 activity and induction of ROS-mediated p38 MAPK signaling pathway in gastric cancer cells could recently be shown [206]. Moreover, Shi and colleagues were the first to report induced apoptosis by ALT (1.5 and 10 μM) in a dose-dependent manner and the inhibition of autophagy by upregulating AP2M1 in acute lymphoblastic leukemia [208]. Only recently, it was found that ALT inhibited the metastatic phenotype and induced the apoptosis in glioblastoma cells by targeting LIMK kinase activity and activating the cofilin/G-actin signaling cascade [210]. Moreover, an anti-cancer effect of ALT treatment on esophageal cancer cells through inhibition of the Wnt/β-catenin signaling pathway was reported [211]. ALT also exerted synergistic anti-tumor effects with other chemotherapeutics [208]. For instance, ALT strengthened the efficacy of epidermal growth factor receptor inhibitors such as erlotinib or afatinib against pancreatic cancer cells [207], as well as cisplatin against lung adenocarcinoma [204]. Furthermore, it enhanced oxaliplatin-induced growth restrain and apoptosis in colon cancer cells [203] and showed synergism with erastin as a potent anti-gastric cancer remedy [206].

To date, many studies continue to be published highlighting the potential of ALT and IAL as novel agents against various ailments, although their pharmacologic activities are unfortunately curtailed due to their low water solubility and low oral bioavailability [212]. However, ALT has been shown to have a rapid onset, without causing significant damage to normal animal tissues and organs in vivo [213].

### 2.7. Sassafras albidum

*Sassafras albidum* (Nuttall) Nees (sassafras) is an aromatic tree, a member of the family Lauraceae and native to the eastern regions of the USA [214]. The summer-green tree reaches a height up to 30 m and it is characterized by a multivariate foliage, with unlobed, single- and triple-lobed leaves growing concurrently on the same twig [215]. Small, yellowish flowers are arranged in umbels, and the small, oval berries (drupes) are deep blue [13] (p. 609). The long lateral roots of its shallow root system develop suckers, which in turn develop their own root system [216]. Thanks to the root sprouts, sassafras expands clonally, and invades abandoned fields in the early stages of plant succession [217]. Almost all parts of the tree (most notably the root bark) are found to be aromatic [215].

Sassafras has been used for centuries by Native Americans and later by European settlers in traditional medicine, and as a flavoring agent as well as a building material, thus holding a significant place in early colonial history [218,219]. It is reported that sassafras tea out of the steeped sweet aromatic young roots has been traditionally used as a remedy against various ailments. Among them, fever, liver discomfort, headache, bronchial congestion, stomach ailments, kidney stones, gout, toothache, arthritis, constipation, and infertility are described [215,218]. In addition to the traditional use as a tea drug for medicinal purposes, sassafras root and root bark are widely known for their EO, a yellow to amber liquid, which is characterized by a unique sweet, and woody flavor [215]. Therefore, the oil and sassafras have been extensively used as flavoring agents for toothpastes, soft drinks (root beer), meat, and in baking, as well as in perfumes, soaps, and commercial cleansers. Furthermore, its use to mask the taste or odor of other unpalatable medicines is reported. The dried root bark has also been utilized for medicinal purposes as a topical antiseptic and disinfectant for root canals in dentistry [215,220,221,222].

According to the literature the root bark contains up to 9% of oil. The oil content from the entire root ranges from 1.0% to 2.0%, and it accounts for less than 1.0% of the root wood [214]. Several publications on the analysis of *S. albidum* EO revealed the presence of at least 20 constituents, with the outstanding predominance of safrole (60% to 95%) Figure 1(28) [214,217,223,224,225]. According to comparative studies on sassafras root bark and root wood EOs from the Netherlands, safrole (61.3–88.8%) was the main compound in all of the tested samples. Further, camphor (1.2–18.4%), methyl eugenol (1.4–5.4%), and 1,8-cineol (0.8–1.2%) were indicated as the most abundant constituents. According to these authors, the hydrodistilled oils were the same in their composition when root bark and root wood originated from the same root source, but with some quantitative differences [214]. Phytochemical analysis of commercial EO of *S. albidum* root bark from Belgrade identified 95.2% of the total constituents, revealing the presence of 28 different constituents. Safrole (82.0%) was found to be the principal compound besides methyl eugenol (3.0%), and α-calacorene (1.1%). Other components were only found in trace amounts less than 1% of total oil [223].

Investigations on the allelopathic influence of *S. albidum* on the environment elucidated a variation in the presence and concentration of terpenes according to the root diameter. The amounts of safrole increased with root diameter, being the main component in roots larger than 2 mm. However, α-phellandrene—predominating in the smallest roots—was found to exert concentration-dependent reduction in radicle growth in *Acer negundo* and *Ulmus americana* [217].

Safrole—also known as shikimol—is a colorless or pale, yellow liquid with a sweet, anise, and floral taste. It therefore has been widely used as a flavor and fragrance agent prior to 1961 [219,221]. *Cinnamomum camphora* L., *Ocotea pretiosa* (Ocotea cymbarum), and *Piper betle* L. are other economic sources of safrole. Small amounts are contained in spices such as anise, basil, nutmeg, mace, and black pepper [215,224]. However, safrole and closely-related compounds such as isosafrole Figure 1(29) and dihydrosafrole Figure 1(30) were recognized as weak hepatocarcinogens in rats and mice and possibly in humans, with safrole appearing to be the primary hepatocarcinogenic volatile constituent of the sassafras root bark oil [226,227]. Moreover, several in vitro and in vivo studies also elucidated safrole to be mutagenic and exert genotoxicity in connection to its hepatotoxicity [219,228]. Henceforth, multiple studies on its carcinogenicity and metabolism have been conducted, as recently summarized by Kemprai and colleagues [219]. According to these authors, the hepatotoxicity of safrole is associated with metabolic bioactivation, leading to the formation of electrophilic metabolites [228,229]. Thus, the carcinogenicity of safrole is believed to be mediated through 1´-hydroxysafrole formation by CYP450 oxidation, followed by sulfonation to an unstable sulfate, which forms DNA adducts [230,231]. The concern of a possible accumulation of DNA adducts upon regular exposure to low dietary levels of safrole due to inefficient repair has recently been strengthened [232].

The FDA were first to ban safrole and sassafras oil as well as isosafrole and dihydrosafrole as food additives and flavoring agents in 1960 [233]. Furthermore, safrole was also categorized as a 2B carcinogen by the International Agency for Research in Cancer (IARC, 1976) [234] and therefore—together with isosafrole and dihydrosafrole—prohibited by the IFRA as fragrance ingredients in 1987. According to IFRA-standards, a total concentration of 0.01% should not be exceeded in any consumer product [22]. Nevertheless, safrole can still be found as a flavor in tobacco products and by-products, such as e-cigarettes, inducing potential harms from inhaling these chemicals and by-products [235]. However, the purified, safrole-free aqueous extract from sassafras root bark is considered safe and is allowed as a flavoring in food [236].

It was found that besides the liver, human buccal tissue is possibly exposed to a high concentration of safrole by oral administration [231]. According to literature data, chewing betel quid (Areca) with *Piper betle* inflorescence as unique ingredient in Taiwan leads to a high concentration of 420 µM (70 µg/mL) of safrole in human saliva [237]. This in turn is associated with safrole-inducted [Ca^2+^]i increases, the formation of stable safrole-DNA adducts, and the enhancement of human oral cancer cells proliferation [238]. This may explain the increased risk of oral squamous cell carcinoma and oral submucous fibrosis in association with Areca chewing [239]. The observed likelihood of periodontal diseases and prevalence of oral gingivalis infections of Areca chewers [240] may also be associated with safrole, which has been significantly found to compromise oral health. Safrole exposure at high concentrations (5 mM and 10 mM) reduced the defensive functions of human neutrophils by inhibiting their bactericidal activity and production of superoxide anion. Oral pathogens including *Actinobacillus actinomycetemcomitans* and *S. mutans* were inhibited in a dose-dependent manner [241]. At 10 mM of safrole, 50% of the phagocytic activity of polymorphonuclear leucocytes (PMNs) was affected, while an exposure up to 5 mM of safrole had no impact. Additionally, the normal activation activity of PMNs was impaired. Briefly, it can be said that the alternation of the defense mechanisms from PMNs in the cervicular area and saliva may promote bacterial colonization and periodontal infection [241,242]. By contrast, these findings also increase the possibility that exposure of PMNs to safrole reduces the inflammatory reaction and reduces the capacity of PMNs to damage tissue [241,242].

However, Ni and colleagues were the first to show that safrole (at 10 µM and attaining a plateau at 100 µM) promoted the expression of pro-inflammatory cytokines, including TNFα, IL-1β, IL-6, and NO in macrophages via NF-κB/IκB pathway and its upstream factor, MAPK family phosphorylation [243]. Further, safrole treatment induced cytotoxicity, genotoxicity, and apoptosis in macrophages through the generation of intracellular ROS and suppression of anti-oxidative enzymes, possibly via Akt (protein kinease B) phosphorylation [244]. In summary, the results obtained elucidated that safrole exerted its toxic effects on different tissues at high concentrations [245].

Lin and colleagues were able to show that safrole became cytotoxic to human osteosarcoma cells at higher concentrations. The researchers stated that safrole’s in vitro [Ca^2+^]i elevating and apoptotic effect in osteosarcoma cells may be physiologically significant in people who consume large amounts of betel quid daily [245]. In human oral squamous cell carcinoma HSC-3 cells, safrole-induced apoptosis through the mitochondria-dependent cell death pathway in vitro and significantly inhibited (40%) tumor size of HSC-3 cells in xenograft tumor cells in vivo [234]. Furthermore, safrole exerted dose-dependent anti-cancer activity against human leukemia HL-60 cells, inducing apoptosis through the ER stress and mitochondria-dependent signaling pathways [246]. Moreover, a high anti-hepatoma effect of safrole has been reported in vitro [247]. However, lower doses of safrole (less than 16 mg/kg) may act as a potent immunological adjuvant, promoting immune response and increasing the activities of macrophages phagocytosis and NK cells’ cytotoxicity in BALB/c leukemic mice [248].

Based on its strong structural versatility, safrole has remained an important synthon for the synthesis of numerous bioactive molecules through small chemical transformations [219,229]. This includes prostaglandin analogues, non-steroidal anti-inflammatory agents, and antithrombotic compounds [249]. Furthermore, piperonyl butoxide—an insecticide synergist used in a wide variety of pesticides [250]—and heliotropine (piperonal)—a fragrance substance and synthon for several drugs, including L-Dopa, tadalafil, and atrasentan—are being synthesized from safrole as a starting material [219]. Only recently, eight new imides with strong antifungal activity against various strains of *Candida* and *Cryptococcus* have been synthesized from safrole [251]. Modified collagen cross-linked with epoxidized safrole (OYBD) was found to exert excellent antibacterial activity against Gram-negative *E. coli*, and Gram-positive *S. aureus*, respectively. Furthermore, increased mechanical and thermal collagen-properties without functionality loss were reported. Therefore, OYBD showed strong potential as a novel modifier for the development of antibacterial collagen-based biomaterials [252]. Apart from that, isolated safrole from *Ocotea odorifera* EO was recently found to possess remarkable antibacterial and antibiotic-modulating properties against multi-resistant efflux pump (EP)-carrying strains. While safrole exerted clinical irrelevant activity against *E. coli* and *P. aeruginosa*, promising effectivity against *S. aureus* (512 µL/mL) could be observed, probably by directly inhibiting the NorA and MepA efflux pumps. Moreover, safrole potentiated the antibacterial activity of norfloxacin up to seven-fold against all the tested strains [253].

*Sassafras albidum* EO was also reported to exert moderate fungitoxicity against various soil-borne pathogens, food storage spoilage fungi, mycotoxins producers, as well as plant, animal, and human pathogens. These include *A. niger*, *A. ochraceus*, *P. ochrochloron*, *P. funiculosum*, *Cladosporium cladosporioides*, *C. fulvium*, *Trichoderma viride*, *F. tricinctum*, *F. sporotrichoides*, *Phoma macdonaldii*, *Phomopsis helianthi,* and *Mucor mucedo*, with similar MICs (5–25 µL/mL) compared with the commercial drug bifonazole (10–20 µL/mL) [223]. Investigations on in vitro fungitoxicity of safrole against phytopathogenic fungi *C. acutatum* and *Botryodiplodia theobromae* revealed low activity. Interestingly, its nitrated derivative, 6-nitrosafrole, demonstrated a fungitoxicity level similar to that displayed by the commercial fungicide carbendazim [254]. Besides its antimicrobial activities, *S. albidum* EO was also evaluated among 92 plant EOs for its potential as an insecticide in the control of *Thrips palmi* in greenhouses. The vapor phase toxicity bioassay indicated potent lethal activity (95%) from sassafras oil and moderate fumigant toxicity (24 h LC_50_ = 32.75%), being 1.9 times more active than the commercial insecticide dichlorvos [255]. Among 16 tested main constituents from rosemary and nutmeg EOs, safrole exerted potential repellent activity—similar to myristicin, and terpinolene—*Dermacentor variabilis* ticks, known as a robust tick capable of vectoring human diseases. The median repellent times caused over a 300 s observation period in a non-human subject screening assay was higher than synthetic diethyltoluamid (DEET) and the parent EOs [256].

However, safrole, isosafrole, and piperonal are also known as the most important precursors of the illicit party drug ecstasy (MDMA; 3,4-methylenedioxymethamphetamine) [215,224]. Therefore, safrole is registered as a List 1 Chemical by the US Drug Enforcement Administration [257]. In this context, some publications conducted comparative analyses of the composition of commercial *S. albidum* EO with the samples of seized *S. albidum* oils from clandestine laboratories considering forensic aspects [224,225].

### 2.8. Saussurea costus

*Saussurea costus* (Falc.) Lipsch. (syn. *Saussurea lappa* (Decne.) C. B. Clarke, Aucklandia lappa, Aucklandia costus Falc.) belongs to the Asteraceae familiy and is commonly known as costus or kuth [258,259]. The medicinal plant grows in the Himalayan region 2500–3000 m above sea level, and it is cultivated in a few states of India due to its encreasing national and international market demand [260]. Through its extensive use, *S. costus* became a tremendously endangered species, and it is thus enlisted in the Endangered Plant Species of Wild Fauna and Flora Appendix I of CITES (Convention on International Trade in Endangered Species of Wild Fauna and Flora) [261]. It is a perennial, strong, and upright herb with a stem up to 1–2 m high and membranous, irregularly toothed leaves, whereby the upper ones are small, and the basal ones very large. The dark bluish purple to black colored, stalkless, rounded, hard flowers are arranged in axillary and terminal clusters of two to five flowers. Costus has cupped and compressed archene fruits, and a double feathery, brown, and curved pappus. The roots are dark brown in color, strouting around 60 cm and characterized by a bitter, sweetish, and pungent taste [262,263].

*Saussurea costus* has been known for 2500 years as an essential medicinal plant, widely used in several indigenous systems of medicine such as Ayurvedic, traditional Chinese, Tibetan, and Unani [259,262]. Especially root parts and their powder, paste, and oil are used alone or in combination with other drugs by indigenous people in various herbal remedies. It is widely known as an important remedy in the treatment of diseases and infections such as inflammation, gastritis, ulcer, diarrhea, and liver diseases, as well as against allergy, throat infection, asthma, epilepsy, joint pain, typhoid, and scalp scabies [259]. In the Himalayan region, root stocks and roots are further applied for skin diseases, rheumatism, toothache, and dysentery [264]. According to the Ayurvedic system of medicine, the root is mainly adiministered to improve complexion, leucoderma, erysipelas, itching, and ringworm [265]. Furthermore, it has been used since ancient times in the cure of cancer in TCM [266]. It has also been claimed to be carminative and stimulant and it is mentioned as a remedy against digestive disorders, premenstrual syndrome, and menopausal symptoms [259]. According to the Unani system of medicine, the root is used as a carminative, as well as an aphrodisiac, anthelmintic, tonic, brain-stimulating, and against diseases of the liver, kidney, and blood [265]. Costus roots are further traditionally used for alleviating pain and swelling [267]. The high medicinal value of *S. costus* and the broad spectrum of biological activities for different *S. costus* extracts are well documented [259,262,268,269].

The oil extracted from the roots—known as costus oil—is pale yellow to brownish in color and characterized by a very strong odor [268]. Thus, costus oil is a very costly ingredient in high-grade perfumes. It blends well with vetiver, patchouli, rose, violet, and sandal wood, and it is used in preparation of hair oil, besides insecticide and insect repellent and as an incense [268,270]. Indigenous people use the oil on joints to gain relief from pain and for its claimed efficacy against paralysis [259], as well as topically against scalp scabies [262]. However, costus oil is responsible for numerous cases of versatile skin reactions, associated with its high content of sesquiterpene α-methylene-γ-butyrolactones [182,271]. Thus, costus root oil—absolute and concrete have been listened in the IFRA-standards as prohibited fragrance ingredients since 2006 [22].

Different phytochemical investigations on the chemical composition of the *S. costus* root EO have revealed a large variability in the relative amounts and composition of the constituents, with recoveries ranging from 0.02 to 3% [272,273]. Besides different eco- and chemotypes, different growing localities, and environmental factors (temperature, relative humidity, irradiance, photoperiod) as well as storage duration may be responsible for this great variance [273,274,275,276]. Several in vitro and in vivo test models have been developed to investigate the pharmacological properties of the *S. lappa* plant. In general, they have deduced many of its activities, such as anti-ulcer, anti-tumor, hepatoprotective, and choleretic, cardiotonic, and immunomodulatory impact [262].

Liu et al. detected 39 constituents (94.78% of the total oil) in the EO of *S. costus* by hydrodistillation of commercial roots from China. Dehydrocostus lactone (DEH) (46.8%) Figure 1(31) and costunolide (COS) (9.3%) Figure 1(32) were registered as major components, followed by 8-cedren-13 ol (5.1%), α-curcumene (4.3%), α-selinene (2.8%), α-ionene (2.5%), and α-pinene (2.5%) with concentrations > 1% [276]. Analyses of the hydrodistilled EO obtained from root samples growing in India enabled the identification of 42 constituents (yield 0.23% *w*/*v*). It was pale yellow in color, with β-costol (13.6%) and δ-elemene (12.7%) detected as the main components. Additionally, α-selinene (5.0%), β-selinene (4.5%), α-costol (4.0%), 4-terpinol (3.4%), elemol (3.2%), α-ionone (3.1%), β-elemene (3.0%), (-)-γ-elemene (2.1%), p-cymene (2.0%), and 2-β-pinene (1.6%) were described [260]. However, the root EO from Saudi Arabia was obtained in a significantly higher yield (3%) than previously reported. Steam distillation of *S. costus* roots revealed COS (52.0%) and elemene (7.2%) as the principal components in the reddish yellow liquid, besides phenanthrenone (3.0%), caryophyllene oxide (2.4%), (*Z*,*Z*)-9,12-octadecadienoic acid (2.1%), and cyclo-hexane (2.1%) [273]. *Saussurea costus* EO from commercial Algerian roots, obtained by SFE, was characterized by 21 identified compounds, accounting for 82.8% of the total oil. DEH was identified as the principal component (55.4%), followed by COS (8.9%), dehydrosaussurea lactone (6.6%), and aplotaxene (4.7%) [277].

The results from comparative studies conducted on the volatile aroma components of hydrodistilled *S. costus* roots from China and Korea indicated a superior quality and quantity of *S. costus* EO from Korea compared to that the oil from China. Thus, the yield of the Korean EOs with 0.02% was about two-fold higher and with 63 aroma components more complex than those from China, with 46 identified constituents. The predominat hydrocarbons in the Korean EO were quantitatively higher than in the Chinese oil, while (7*Z*,10*Z*,13*Z*)-7,10,13-hexadecatrienal (21.2–23.3%) was identified as the main component in both oils [275]. The EO from the Uttarakhand (Himalayan region) yielded—similar to the reports from Korean oil—0.02% (*v*/*w*) but was more volatile in flavor compounds and higher percentage of total peak area compared with EOs from Korea and China. However, (7*Z*, 10*Z*, 13*Z*)-7, 10, 13-hexadecaterinal (25.5%) was also identified as the main component in the Indian sample, followed by DEH (16.7%), elemol (5.8%), valerenol (4.2%), vulgarol B (3.1%), (E)-β-ocimene (2.3%), β-elemene (2.1%), γ-costol (1.8%), and terpinen-4-ol (1.6%). Overall, the oil profile of the Indian EO was comparatively less complex with 35 identified aroma compounds [278].

Investigations into *S. costus* root EOs from Garhwal (Himalaya) collected at different harvesting periods revealed similar oil contents, ranging from 0.02% to 0.04%, strongly differing from previous reports in compounds and concentration. Dihydroneoclovene, glaucyl alcohol, DEH, alloaromadendrene, glaucyl alcohol, and hexadecanoic acid have been reported as the main ingredients. DEH (1.4–8.9%)—with the highest concentration in three-year-old plants collected in April and December—was found to be two times lower in concentration compared to previous publications. Thus, it has been convincingly illustrated that, biodiversity, harvesting period (month and year), and plant age also have a strong impact on the chemical composition of costus root oil. Moreover, variations in the observed moderate radical scavending and reducing power activities of costus oil have been observed according to the harvesting period, being the highest in samples collected during summer [272].

Despite this large diversity of its chemical contents, several studies have shown that costus root oil exerts numerous biological activities, mainly contributed to its principal components COS and DEH and their α-methylene-γ-butyrolactone moiety [181]. Both sesquiterpene lactones have been extensively studied, indicating anti-oxidative, anti-inflammatory, anti-cancer, antiallergic, antidiabetic, antimicrobial, bone remodeling, neuroprotective, and hair growth-promoting properties [181,279]. Only recently, it could be shown that isolated DEH (5–20 μM) induced caspase-dependent apoptosis in human endometriotic cells. Furthermore, the expression of inflammatory/pain-related factors in human endometriotic cells as well as the alternative activation of macrophages stimulated by them were already suppressed by this compound at lower concentrations (0.5, 1, and 2 μM). Thus, the traditional claim of *S. costus* in treating endometriosis activities in Asian systems of medicine has been strengthened [280]. Zhou et al. were the first to report the anti-inflammatory potential ofDEH to treat ulcerative colitis in mice. Accordingly, the protective mechanism of DEH may be involved in reducing inflammation and improving colorectal barrier function via downregulating the IL-6/STAT3 signaling [281]. The anti-inflammatory effect of COS has also recently attracted strong attention. Thus, COS inhibited microglia-mediated neuroinflammation through targeting directly CDK2-dependent AKT/IKKβ/NF-_k_B signaling pathway. According to the researchers, COS might be a potential anti-neuro-inflammation agent and CDK2 a novel therapeutic target for neuroinflammatory diseases [282].

The traditional usage of *S. costus* in alleviating pain led to the evaluation of the antinoceciptive efficacy of costus oil formulations. Thereby, in all tested systems a dose- and time-dependent analgesic activity (both slow- and fast-onset in manner) could be shown. It is stated, that the demonstrated activity of costus oil may be related to its interaction with opioid receptors and involvement of peripheral analgesia [267]. In this context, topical costus oil was further examinated as a palliative treatment in patients suffering from chemotherapy-induced peripheral neuropathic symptoms. Based on this preliminary study, the use of costus oil was feasible and acceptable in such patients. Moreover, the neuropathic pain VAS score significantly decreased in the verum group (26.5%). Future studies are needed [283].

Apart from that, *S. lappa* EO in its free and encapsulated form was recently studied for several properties. Thereby, an in vitro anti-inflammatory (reduction in metalloprotease MMP-9 enzyme activity and RNA expression of inflammatory cytokines: TNF-α, GM-CSF, and IL-1β), and anti-Alzheimer activity (IC_50_ = 25.0 and 14.9 μg/mL against acetylcholinesterase (AChE) and butyrylcholinesterase, respectively) were reported for *S. lappa* EO-loaded polymethyl methacrylate based nanoparticels. Moreover, a strong antidiabetic effect, with IC_50_ = 22.9 and 75.8 μg/mL against α-amylase and α-glucosidase, respectively, could be shown. No cytotoxic effect at 25 μg/mL was observed [277].

The anti-cancer activities of COS and DEH have led to inceased interest in researchers, highlighting strong potential therapeutic effects against a wide range of cancer types, particularly breast cancer and different types of leukemia. Li and colleagues summarized the anti-tumor activities and related action modes of these two sesquiterpenes, including cell cycle regulation, apoptosis induction, telomerase activity inhibition, anti-metastasis and invasion, anti-angiogenesis, and multi-drug resistance reversion activities [181]. However, Peng and colleagues were able to show that the anti-breast cancer efficacy exerted by the volatile oil from *S. costus* was stronger and associated with lower side effects than isolated COS and DEH alone in vivo, probably due to a multiple-targets mechanism. Furthermore, the EO not only inhibited the growth of tumors, but also maintained the weight and vitality of MCF-7 xenograft mice. On the contrary, DEH was more toxic than COS, while the combination treatment of both components exerted synergistic anti-cancer effects in breast cancer in vivo and in vitro by inducing apoptosis with regulation of the c-Myc/p53 and AKT/14–3-3 signaling pathways [284,285,286]. These findings are congruent with those from another study conducted on the efficacy of *S. costus* EO and isolated COS and DEH against liver cancer. The potent anti-hepatocellular carcinoma activity of *S. costus* EO by regulating the EGFR pathway was found to be stronger than pure COS and DEH. This efficacy was also higher than the synergistically acting combination treatment of isolated COS and DEH [266]. Apart from that, recent results revealed that COS inhibited colorectal cancer cell proliferation and triggered apoptosis in vitro and in vivo by targeting the AKT-MDM2-p53 signaling pathway. Consequently, this compound was found to be worthy for further exploration in preclinical and clinical trials [287]. Moreover, COS has emerged as a potential tumor-specific candidate in skin cancer treatment. The anti-cancer activity of COS on human epidermoid carcinoma cells included the induction of apoptosis and suppression of cell proliferation and survival via various signaling pathways [288].

Regarding the antimicrobial properties, early studies have demonstrated an activity of the EO obtained from *S. costus* root against *S. aureus*, *Aerobacter aerogenes*, *Shigella flexneri*, *P. vulgaris*, *K. pneumoniae*, and *P. aeruginosa* [289]. Assessing the oil’s antistaphylococcal impact, strong in vitro activity with MICs ranging from 0.15 to 0.6 μL/mL could be shown. Furthermore, subinhibitory concentrations of costus oil reduced pathogenicity, contributing to exotoxins production (α-toxin, enterotoxin A and B, and TSST-1) in MSSA and MRSA, respectively. Hence, costus oil may be a potential antibacterial agent with therapeutic value as well as a novel food preservative against *S. aureus* growth [290]. Dose-dependent antifungal activity of costus oil against *C. albicans* could be shown, as well as antibacterial activity against *E. coli, P. aeruginosa*, *S. aureus*, and *B. subtilis* [273]. Furthermore, isolated DEH exhibited MICs of 2 and 16 µg/mL against *M. tuberculosis* and *M. avium* [291]. However, drug-resistant *M. tuberculosis* clinical isolates were found to be more susceptible to isolated COS and DEH in combination by exerting a synergistic activity [292]. Investigations on sesquiterpene lactones indicated remarkable antifungal activity of COS and DEH against *Cunninghamella echinulata*. EC_50_ values of 6 μg/mL were close to those from ketokonazole (1.5 μ/mL) used as an antifungal reference drug. Accordingly, α-methylene-γ-lactone and a relatively low polarity might be molecular requirements for the antifungal activity of sesquiterpene lactones [293].

Additionally, COS and DEH inhibited the expression of hepatitis B virus surface antigen (HBsAg) in human hepatoma Hep3B cells (IC_50_ of 1.0 and 2.0/μM). Remarkable suppression was also observed in human hepatoma cell line HepA2 derived from HepG2 cells. Hence, COS and DEH are suggested as potential anti-HBV drugs in the future [294].

Furthermore, quite promising potential in the control of two main species of mosquitoes—*Ae. albopictus* and *Ae. aegypti*—could be demonstrated [276]. *Aedes albopictus*—the Asian tiger mosquito—showed high larval susceptibility to costus oil, DEH, and COS. Both isolated compounds exhibited almost the same larvicidal toxicity compared with the commercial insecticide cloropyrifos (LC_50_ values of 2.34 and 3.26 μg/mL, respectively), while costus oil was seven times less toxic with LC_50_ = 12.41 μg/mL [276]. Manzoor et al. evaluated the larvicidal activity of five different EOs against 3rd instar larvae of *Ae. aegypti* and *Culex quinquefaciatus* larvae, indicating LC_50_ values of 128.89 and 141.43 ppm for *S. costus* EO, respectively [295]. Although the results obtained were quite promising, further investigations are necessary to establish their human safety and environmental safety [276].

In addition to the aforementioned biological properties, Huntose et al. reported that the inhalation of costus EO by women in labor minimized the symptoms related to pain during the course of labor. Moreover, anxiety, apprehension and related symptoms as well as a mild sedation were alleviated, without exerting any adverse effect for mother and foetus [296]. However, costus oil should be avoided during pregnancy and lactation, due the anti-angiogenetic activity from COS and DEH [297].

COS and DEH were further found to exhibit CNS-depressant activity by increasing hexobarbital-induced sleeping time and reducing body temperature, nociception spontaneous locomotor activity [298]. The antidepressant effect of *Aquilaria sinensis* and *S. costus* EO, a traditional herbal pair in TCM to treat depression has been strengthened only recently. Inhalative administration revealed antidepressant activity in chronic unpredictable mild stress rats by regulating the HPA axis and levels of monoamine and cholinergic neurotransmitters, immunity and gastrointestinal function [299]. It is stated that the comprehensive effects on the central nervous system may, among others, be related to DEH from *S. costus* EO, and agarospirol in *A. sinensis* EO. The two compounds are known as analgesics and potent antagonists of dopamine D_2_ and serotonine 5-HT_2A_ receptor binding [299,300].

### 2.9. Valeriana officinalis

*Valeriana officinalis* (L.) s.l., also known as valerian, is a herbaceous perennial plant from the Caprifoliaceae family [301], that mainly occurs in the temperate zone of the northern hemisphere on sporadically wet habitats [302]. Valerian is a very polymorphic collective species, comprising naturally occurring sub-species that differ from each other by their degree of ploidy [303]. The stem of the shrub attains a height of 30–150 cm, depending on the species, and is characterized by a highly variable aerial habit, with usually imparipinnate leaves, and variably serrated leaflets. The white to pink inflorescences develop in the second year after vernalization. The rootstock forms a dense meshwork of thin roots [302], [13] (p.692), with a clonical, dull brown colored rhizome and many long strout attached roots [304]. While the fresh root is odorless, dried roots smell distinctly unpleasant, comparable with valeric acid and camphor [305,306]. The taste is described as slightly bitter and spicy [306].

For medicinal purposes, the entire root system including the rhizome is used [307]. From ancient times, *V. officinalis* has been used to treat nervine and sedative in hysteria, epilepsy, and sedative in nervous anxiety [301]. It is widely used against excitability, hypochondriasis, migraine, cramp, intestinal colic, rheumatic pains, etc. [308]. Valerian preparations have further been shown to encourage sleep, improve sleep quality, and reduce blood pressure [309]. Additionally, the root is known for its mild anodyne, hypnotic, carminative, and hypotensive properties [301]. Sleep promotion and anxiolytic effects are the major therapeutic benefits expected from *V. officinalis* herb [308,309], and thus have been a major research focus, as extensively reviewed only recently [309]. Neverthless, those responsible components and underlying mechanisms remain obscure [310]. Valerian can be further useful in treating obsessive-compulsive disorder, cognitive dysfunction, menopausal hot flashes, as well menstrual problems [309]. According to the Euopean Medicine Agency (EMA), valerian is considered relatively safe and well-tolerated. Gastrointestinal symptoms (e.g., nausea, abdominal cramps) are noted as undesirable effects [311]. Dried valerian roots alone or combined with other crude herbs are also widely used as natural repellents of insects, pests, and some rodents. Especially to fend off cockroaches homes and skunks from golf fields and home gardens [312]. The EO or extracts from *V. officinalis* are used in the formulations of personal care products, cosmetics, aromatherapy, and veterenary practices [312]. It was further used as perfume in 16th century [313]. As cigarettes flavoring, valerian oil is reported to reduce offensive odor, to make tobacco smoke smooth and fine, and to improve smoking quality of the cigarettes [314]. Valerian root oil is not restricted by the standards of the IFRA [22].

The content of *V. officinalis* EO from roots and rhizomes, either wild grown or cultivated, has been reported to vary from 0.1% to 2.8% [303]. According to European Pharmacopeia the minimum content of EO must be 4 mL·kg^−1^ expressed on a dry root weight. Furthermore, >0.17% valerenic acids are requested [307]. Oxygenated monoterpenes, monoterpene hydrocarbons, oxygenated sesquiterpenes, and sesquiterpene hydrocarbons were registered as the most abundant constituents [304,315]. Several publications on valerian root oils clearly indicated remarkable compositional differences, according to various geographic origins. It is further known, that volatile oil production and quality variations of *V. officinalis* root/rhizome are largely associated to genotype and climate conditions [316]. Moreover, based on the principal compound of the EO, four chemotypes can be distinguished within the species *V. officinalis*. This includes the valeranone Figure 1(33), valerianol Figure 1(34), cryprofaurinol Figure 1(35), and valerenal Figure 1(36) type [303].

Out of 15 studied valerian root samples from different European countries (Belgium, Czech, Estonia, France, Germany, Greece, Hungary, Latvia, Lithuania, Moldova, Russia, Scotland, Ukraine), nine belonged to the bornyl acetate/valerenal chemotype. The distilled EO recoveries ranged from 0.19% to 1.16%, with bornyl acetate (2.9–33.7%), α-fenchene (0–28.3%), valerianol (0.2–18.2%), valerenal (tr-15.6%), isovaleric acid (0–13.1%), camphene (0–11.1%), valeranone (0.5–10.9%), valerenic acid (0–9.8%), sesquiterpene alcohol C (tr-8.0%), spathulenol (0.3–7.3%), and allo-aromadendrene (0.3–6.9%) as the basic oil constituents among 86 identified compounds (> 90% of the total oil) [317]. Also, the steam distilled EO from commercial *V. officinalis* roots/rhizome grown in China, with 39 identified constituents, was mainly characterized by bornyl acetate (46.9%) as the principal ingredient, although the other most representative compounds differed, apart from camphene (13.9%). They included *trans*-valerenyl acetate (13.2%), mirtenyl acetate (3.9%), α-pinene (3.4%), and β-pinene (2.8%) [304]. Similarly, another Chinese (Hubei Province) *V. officinalis* root EO, obtained by hydrodistillation, was also dominated by bornyl acetate (48.2%), camphene (13.8%), β-pinene (2.8%), α-pinene (2.7%), borneol (2.1%), and myrtenyl acetate (2.0%). With 17 identified compounds (80.0% of total oil), it was less complex in its composition [318]. Conversely, patchoulol (16.8%), α-pinene (14.8%), β-humulene (8.2%), α-bulnesene (7.1%), and d-limonene (6.6%), were the most abundant constituents among 20 identified compounds (88.11% of the total oil) of hydrodistilled valerian root/rhizome EO also from China (Henan Province). Furthermore, bornyl acetate (6.7%) and camphene (6.5%) were registered in comparatively much less amounts [315]. Regarding the EO composition of *V. officinalis* roots cultivated in the USA, steam distillation also afforded an EO with a completely different profile. The main constituents were found to be juniper camphor (18.7%) and β-gurjunene (15.3%), follwed by d-guaiene (9.5%), α-gurjunene (9.5%), farnesol (6.5%), α-panasinsene (5.8%), and patchouli alcohol (5.7%). In total, 16 constituents were registered, accounting for 92.66% of the total oil [319]. However, the yellowish root/rhizome EO from *V. officinalis* growing wild in Serbia, obtained by hydrodistillation (1.88% yield), can be considered as a valerianol-chemotype. The EO, consisting of 53 identified constituents (90.7% of total oil), was outstandly dominated by valerianol (57.3%). Other compounds with amounts >2% were bornyl acetate (11.3%), α-fenchene (4.5%), longiborneol acetate (2.2%), and valencene (2.1%) [320].

However, the EO of cultivated valerian roots from Iran, with 69 identified compositions, matched the literature proposed valerenal chemotype, with valerenal (12.9%) as the major constituent, followed by camphene (11%), bornyl acetate (10.1%) borneol (6.6%), α-fenchene (6.1%), valeranone (5.8%), and germacrene-B (2.4%). The EO content, obtained by hydrodistillation, was 1.65% [321]. Conversely, steam distilled valerian root oil from Myanmar consisted of transcalamenene, methylsterate, α-guaiene, *trans*-ligustilide, *cis*-ligustilide, hexadecanoic acid, and methyl ester [305].

Apart from compositional variations of valerian EO regarding various geografic origins, remarkable differences were demonstrably also related to cultivar type, plant age, harvesting time and the year of cultivation [312,322]. Thus, the hydrodistillation of 8- and 14-month old plants, grown under certified organic commercial cultivation in Northwestern United States yielded 0.67% and 0.87% EO from the cultivar (cv.) Select. The recoveries from cv. Anthose ranged from 0.97% to 1.1% EO. All EOs were characterized by a bitter taste and valerian-like smell. Neverthless, the greenish-yellow collored EO from cv. Select had a heavy typically moldy, dirty socks, valerian smell. The EO from cv. Anthose was light green in color, with a light aromatic odor. Regarding the chemical composition, 43 constituents were detected from cv. Select, with valerenal (11.2–14.0%), (−)-bornyl acetate (10.7–12.7%), 15-acetoxy valeranone (7.1–8.9%), valerenic acid (5.0–5.8%), and camphene (4.9–5.7%) as the most abundant constituents. However, valerenal (12.3–13.3%), (−)-bornyl acetate (9.2–9.5%), camphene (7.0–7.2%), α-humulene (7.4–8.5%), 15-acetoxy valeranone (5.3–5.7%), valerenic acid (2.0–5.9%), and gurjunene (4.2–5.2%) were the major components from the more complex cv. Anthose EO (53 identified constituents). In this context, also plant developmental-dependent increases of valerenal, valerenic acid, and α-humulene have been observed [312]. According to another study, higher amounts of valerenic acids could be obtained in the second year of cultivation over the period from full bloom (0.278%) to fall rosette phase (0.316%) [322].

Moreover, seasonal changings are known to influence the content of the EO, valerenic acid and derivates in *V. officinalis* root/rhizome [323,324]. Thus, recent comparative studies on EO composition, content, and sesquiterpenic acid content in valerian roots collected wild and harvested in organic farming system elucidated accordingly variations. A total of 37 compounds were identified in hydro-distilled EO of five valerian populations from Latvia. Bornyl acetate, valerianol, valeranone, intermedeol, camphene, myrtenyl acetate, agarospirol, and γ-eudesmol were reported as the main constituents [324]. The contents of EO and valerenic acids demonstrably also vary according to the rootstock-part. High contents were located in the adventitious roots, respectively, followed in descending order by the lateral roots and the rhizomes. Rhizomes had distinctly lower contents of EO and valerenic acids in comparison to the roots. Generally, these observations underline the importance of careful harvesting and preparation of valerian by hand, as the partial lost of the adventitious and lateral roots during the harvesting, cleaning and drying process resulted in drug yield losses and that in turn degradation of EO recoveries [325]. Moreover, careful handling avoids the damage of the root surface and leads to higher EO contents, as histological imaging techniques elucidated that large number of oil droplets (an average 43% of total oil droplets) are located close to the valerian root surface. The remaining oil droplets are located in the inner regions (parenchyma), with varying density gradients from the inner to the outer regions depending on genotype, root thickness, and harvesting depth. Further studies are needed to evaluate the relationship between the EO droplet density and EO content [326]. However, contrary to previous assumption [325], no linear relationship between oil drop density and root thickness could be derived [326]. Despite the harvest process, sowing time and planting density were also found to significantly affect EO yield and percentage. Accordingly, late-planted and low planting density increased the EO content and composition in valerian [327].

In order to improve the consistency of the raw material and to obtain higher root yields and oil production in valerian, the impact of various cultivation systems was assessed. It could be noted, that the EO concentration was the highest in the floating system as compared with the other growing systems, without significant difference among the aeroponics, growing media, and soil systems. Moreover, growing valerian plants in the floating system induced comparatively the highest production of bornyl acetate (56.5% higher), while the production of valerenal was the highest in the soil system. Results also indicated positive correlation between photosynthesis and EO content [328]. With regard to productivity optimisation in a scenario of drought and climate change, several publications evaluated the effect of drought stress on qualitative and quantitative traits of *V. officinalis* [329,330]. Root growth decreased with increasing water shortage, while the EO content was positively affected by moderate drought stress treatment (50% available water content). However, very severe drought stress conditions (30% available water content) led to reverse trends. Although the exogenous application of polyamines (growth regulators) was ineffective on EO accumulation, it alleviated reverse effects of stress on plants by increasing root growth and consequently improving EO yield [330]. These results are in line with another study, where reduction of valerian EO content induced by water stress was compensated by increasing root yield as a result of bio-fertilizer (rhizospheric bacteria and arbuscular mycorrhizal fungi) application [329].

Qualitative and quantitative variations in the chemical composition of *V. officinalis* EO also depend on drying procedures. Steam distillation of fresh valerian roots from British Columbia, Canada, afforded 0.17% (*v*/*w*) of a pale yellow-green oil, while the recovery of dried roots was 0.50% (*v*/*w*) of a yellow oil. In total, 61 components were detected comprising 78.7% and 76.2% of the EO composition, respectively. Thereby, an increase in borneol and sesquiterpene hydrocarbons in dried root oil, in comparison to fresh roots was found, as well as a higher content of isovaleric acid (13.0%), compared to 0.2% in fresh roots. The latter might be related to the enzymatic hydrolysis of isovaleric esters upon drying. Same study also showed the impact of distilling procedures on the chemical composition of the EO obtained from dried roots, and consequently the possibility of content-modification. Thus, the last of three oil fractions revealed an eight-fold higher content of oxygenated sesquiterpenes, accounting for key components [331].

Despite from these factors, analytic results on the chemical composition of wild grown *V. officinalis* roots from Iran, revealed significant compositional differences in the EOs isolated by two different methods. Thus, 35 compounds varying from 86.1% to 95.1% of total oil were identified from the SFE- extracted oil with isovaleric acid (18.7–41.8%) as the main compound. The EO obtained by hydrodistillation with 47 identified components representing 89.3% of total oil, was more complex and dominated by bornyl acetate (11.6%). The hydrodistilled EO was further characterized by valerenic acid (8.0%), (*Z*)-valerenyl acetate (7.9%), and acetoxyvaleranone (7.6%), followed by *E*-caryophyllene (5.1%), spathulenol (4.7%), valerenol (4.3%), allo-aromadendrene (4.1%), kessane (3.3%), epi-α-cadinol (2.8%), dehydro-aromadendrene (2.7%), and valerenal (1.5%). Comparing the oil recoveries, hydrodistillation yielded 0.21% of EO, while SFE-extracted contents ranged from 1.84–4.90% [332].

The potential role of valerian species in traditional medicine for the treatment of neurological disorders led to several phytochemical studies that focused on the biological and pharmacological properties [309]. Studies on the inhalative effect of various odorants on the sleep-wake states in rats, revealed a significant alteration of the phenobarbital-induced sleeping time (+41%) by valerian inhalation, as well as remarkable shortening in sleep latency (−34%) and prolongation of total sleep time (+18%). The additional conducted γ-aminobutyric acid (GABA) transaminase assay, demonstrated the decrease in enzyme activity and enhancement of GABA activity by valerian EO [333]. Due to its sleep prolonging effects, valerian, among others, was also selected for an investigation studying the hypothesis that certain odorants can mitigate the effects of stress on skin immune reactions. Although *V. officinalis* EO inhalation showed only a weak effect on stress-induced suppression of contact hypersensitivity on mice, it caused the downregulation of stress-induced plasma corticosterone levels [334]. A comparative double-blind, randomized, placebo-controlled study on the anxiolytic effects among hospitalized patients with coronary artery disease elucidated that the oral intake of both, lavender EO (80 mg) as well as valerian EO (400 mg) was as effective as oxazepam in alleviating anxiety among patients [335]. Pain reduction is also related to the reduction of pain and labor duration, which in turn results in a good experience of delivery. In this context, valerian (1.5% with olive oil) aromatherapy had better impact compared to lavender (1.5% with olive oil) on reduction of active phase among nulliparous women [336].

In claim of the traditional use as antidepressant, the biological effects of *V. officinalis* EO on the nervous system was assessed only recently. Results indicate concentration-dependent AChEinhibitory activity (IC_50_ = 127.30 µg/mL), and decrease in the spontaneous electrical activity of in vitro corticonal neuronal networks until the complete loss of activity. Compared to the EOs of *Nardostachys jatamansi* and *V. jatamansi*, which are often fraudulently used as adulterants, *V. officinalis* EO exerted the second strongest inhibition, while the EO of *N. jatamansi* induced throughout the highest inhibitory activity [304]. Previous studies evaluated the inhalative effect of *V. officinalis* EO on autonomic nerve activity in depressed as well as healthy subjects. It was found that valerian inhalation stimulated parasympathetic activity in both normal healthy and depressed subjects, while no apparent stimulatory effect on sympathetic activity was observed. As depression is associated with increased sympathetic nervous activity and decreased parasympathetic nervous activity, aromatherapy might have a potentially powerful effect on autonomic nervous function in depression [337]. Valerenic acid, valerenal, and valeranone have been reported to play a significant role in the depressive action of the valerian EO [338].

Although the underlying mechanisms need to be further studied, *V. officinalis* root EO was moreover found to possess a broad spectrum of antibacterial activity. This includes efficacy against Gram-positive *S. aureus* and *S. haemolyticus*; and Gram-negative *Agrobacterium tumefaciens*, *E. coli*, *P. lachrymans*, *S. typhimurium*, and *Xanthomomonas vesicatoria* (MICs ranging from 62.5 μg/mL to 200 μg/mL, and IC_50_ values from 40.00 μg/mL to 144.11 μg/mL). Undereneath them, bacteria such as *A. tumefaciens*, *S. haemolyticus* and *B. subtilis* were the most susceptible to valerian EO, with IC_50_ values of 40.00 μg/mL, 47.37 μg/mL and 48.74 μg/mL, respectively [315]. Evaluating the antibacterial properties of valerian root EO against various foodborne pathogens, *L. monocytogenes* reaveled to be the most sensitive, followed by *B. cereus*, and *Shigella sonnei*. Furthermore, weak activity of the EO against *S. enterica*, *S. typhimurium*, *S. aureus*, *S. epidermidis*, and *S. intermedius* was shown [319]. The antimicrobial properties of *V. officinalis* root EO also included moderate antifungal activity on amphothericin-resistant *C. albicans* growth and *Magnaporthe oryzae* spore germination with IC_50_ values of 165.74 μg/mL and 142.59 μg/mL, respectively [315]. However, analytic results strengthen the assuption that the inhibitory activity of *V. officinalis* root EO depends on biological origin (cultivar) and plant developmental stage, which in turn lead to significant differences in the chemical profile of the EOs. Thus, the above mentioned cr. Select exerted strong antimicrobial effects against *A. niger*, *E. coli*, *S. aureus*, and *S. cerevisiae*, whereas low or no activity against all test microbes, including *P. aeruginosa* was found for cr. Anthose [312].

Additionally to its antimicrobial properties, the root EO of *V. officinalis* is also known for its insecticidal potential, although reports are scarce. Valerian EO possessed a significant fumigant and a moderate contac toxicity against the booklice *L. bostrychophila* (LC_50_ = 2.8 mg/L air and LD_50_ = 50.9 μg/cm^2^, respectively), and a notable contact efficacy on the red flour beetle *Tribolium castaneum* (LD_50_ =10.0 μg/adult). Both pest species showed almost the same leveled sensitivity to fumigation and contact toxicity exerted by the EO main constituents bornly acetate and camphene (LC_50_ = 1.1, 10.1 mg/L air and LD_50_ = 32.9, 701.3 μg/cm^2^ for *L. bostrychophila*; > 126.3, 4.1 mg/L air, and 66.0, 21.6 μg/adult for *T. castaneum*), with camphene as the much more toxic component. Regarding the repellent properties, valerian EO generally pronounced stronger repellency than the extracted main constituents, being more suitable to control the red flour beetles than booklice. Interestingly, the latter revealed to be attracted by camphene, while the red flour beetle was repelled moderately by bornyl acetate [318]. Further studies, conducted by the same research group, demonstrated that attractancy generally was more likely to occur at low concentrations. Thereby, the toxicity and repellent efficacy from EO and SFE extract of four Valerianaceae (*V. officinalis* L., *V. officinalis* L. *var*. *latifolia* Miq., *V. jatamansi* Jones, and *N. chinensis* Bat.) against *L. bostrychophila, T. castaneum* and the cigarette beetle *Lasioderma serricorne* has been evaluated. The strongest contact toxicity to red flour beetle was exerted by *V. officinalis* EO (LD_50_ = 10.0 μg/adult), while the booklouse was outstandingly susceptibily to *V. officinalis var. latifolia* EO (LD_50_ = 40.2 μg/cm^2^). *V. jatamansi* EO showed the highest efficacy against the booklouse (LD_50_ = 40.2 μg/cm^2^). However, the contact toxicity of SFE extracts against the three storage-product insects was relatively weak. An additionally conducted evaluation of binary camphene and bornyl acetate mixtures for the contaxt toxicity to the red flour beetle indicated an additive effect in the natural proportion of *V. officinalis*. Moreover, synergism was found in the natural proportionof *V. officinalis var. latifolia* [339]. In another study potential fumigant toxicity against the sweet potato whitefly, *Bemisia tabaci*, was screened. Thereby no toxicity from valerian EO was found [340]. However, *V. officinalis* root EO demonstrated potential as acaricide, exerting corresponding effects against *Tyrophagus putrescentiae* adult, *Haemaphysalis longicornis* larva, and *H. longicornis* nymph (LD_50_ = 28.01, 178.26, and 207.98 µg/cm^2^, respectively) [319].

Despite its antimicrobial and insecticidal properties, *V. officinalis* root EO was also evaluated for its antioxidant activity [341,342]. Using three complementary tests, namely the 1,1-diphenyl-2-picrylhydrazyl (DPPH) free radical scavenging- (IC_50_ = 493.40 μg/mL), β-carotene bleaching- (IC_50_ = 181.18 μg/mL), and ferrozine Fe^2+^ complex formation assay (IC_50_ = 235.44 μg/mL), a moderate antioxidant activity of valerian root EO could be shown [315]. Comparing the antioxidant properties of microwave-assisted valerian EO and an oil obtained by steam distillation, strong and concentration dependent scavenging ability to DPPH free radicals and hydroxyl free radicals were found for both oils. However, obvious differences were noticed, with microwave-assisted valerian oil beeing the more potent antioxidant [342].

## 3. Conclusions

In this review, we have attempted to provide an overview of the chemical composition of selected aromatic roots and the research conducted on the main bioactive constituents linked to their various biological activities. In summary, these results strengthen the ethnobotanical value of aromatic roots and elucidate their potential as an important source of remarkable compounds with a diversity of molecular structure and biological activity. However, further efforts are needed to fully evaluate the biological activities, their mode of action, and possible toxicological effects to establish human and environmental safety. Moreover, further studies should focus on plant cultivation and EO standardization because chemical composition within a species and consequently biological activity may strongly vary according to different factors.

## Data Availability

Not applicable.
